# Bayesian Network Modeling Applied to Feline Calicivirus Infection Among Cats in Switzerland

**DOI:** 10.3389/fvets.2020.00073

**Published:** 2020-02-26

**Authors:** Gilles Kratzer, Fraser I. Lewis, Barbara Willi, Marina L. Meli, Felicitas S. Boretti, Regina Hofmann-Lehmann, Paul Torgerson, Reinhard Furrer, Sonja Hartnack

**Affiliations:** ^1^Department of Mathematics, University of Zurich, Zurich, Switzerland; ^2^Independent Researcher, Utrecht, Netherlands; ^3^Clinic for Small Animal Internal Medicine, Vetsuisse Faculty, University of Zurich, Zurich, Switzerland; ^4^Clinical Laboratory, Department of Clinical Diagnostics and Services, Vetsuisse Faculty, University of Zurich, Zurich, Switzerland; ^5^Center for Clinical Studies, Vetsuisse Faculty, University of Zurich, Zurich, Switzerland; ^6^Section of Epidemiology, Vetsuisse Faculty, University of Zurich, Zurich, Switzerland; ^7^Department of Computational Science, University of Zurich, Zurich, Switzerland

**Keywords:** feline calicivirus, reproducible research, good modeling practice, graphical model, multivariable analysis, risk factor analysis, Bayesian network

## Abstract

Bayesian network (BN) modeling is a rich and flexible analytical framework capable of elucidating complex veterinary epidemiological data. It is a graphical modeling technique that enables the visual presentation of multi-dimensional results while retaining statistical rigor in population-level inference. Using previously published case study data about feline calicivirus (FCV) and other respiratory pathogens in cats in Switzerland, a full BN modeling analysis is presented. The analysis shows that reducing the group size and vaccinating animals are the two actionable factors directly associated with FCV status and are primary targets to control FCV infection. The presence of gingivostomatitis and *Mycoplasma felis* is also associated with FCV status, but signs of upper respiratory tract disease (URTD) are not. FCV data is particularly well-suited to a network modeling approach, as both multiple pathogens and multiple clinical signs per pathogen are involved, along with multiple potentially interrelated risk factors. BN modeling is a holistic approach—all variables of interest may be mutually interdependent—which may help to address issues, such as confounding and collinear factors, as well as to disentangle directly vs. indirectly related variables. We introduce the BN methodology as an alternative to the classical uni- and multivariable regression approaches commonly used for risk factor analyses. We advise and guide researchers about how to use BNs as an exploratory data tool and demonstrate the limitations and practical issues. We present a step-by-step case study using FCV data along with all code necessary to reproduce our analyses in the open-source R environment. We compare and contrast the findings of the current case study using BN modeling with previous results that used classical regression techniques, and we highlight new potential insights. Finally, we discuss advanced methods, such as Bayesian model averaging, a common way of accounting for model uncertainty in a Bayesian network context.

## 1. Introduction

Risk factor analysis is often the primary goal of epidemiological studies. When the disease system under study is complex, there are likely many interdependent variables, including multiple interdependent outcome variables. Novel multivariate modeling approaches, such as Bayesian network (BN) modeling, may potentially reveal new epidemiological insights compared to classical statistical approaches (1) when applied to complex disease system data. We present an introduction and guide to BN modeling with complex epidemiological data and provide a case study analysis using animal welfare data. Animal welfare is an intrinsically multi-dimensional concept that cannot be measured directly. Comin et al. (2) included three animal-based welfare indicators: feather condition, mite infestation, and flock mortality. They considered two environment-based welfare indicators: the lightning quality of the barns (i.e., the quality of the lamps within the barns, whether the barns have windows, and whether they are automatically or manually regulated) and the air quality. A typical approach for dealing with multiple outcomes in animal welfare studies is to construct a composite score as the response variable and run a regression analysis. A disadvantage of this approach is that we may lose valuable insights by reducing the different welfare outcomes into a single dimension/outcome variable. Ideally, we want to retain all the richness of the original data. Rather than create a composite variable, we can instead keep all the original outcome variables by using a graphical modeling approach, and the particular type of graphical modeling methodology we consider here is Bayesian network modeling. With the increasing availability of data and the need to understand and explain ever more complex epidemiological systems, knowledge of how to effectively apply new multivariate methods, such as BN modeling may be increasingly relevant for veterinary epidemiologists.

**Figure 1:**
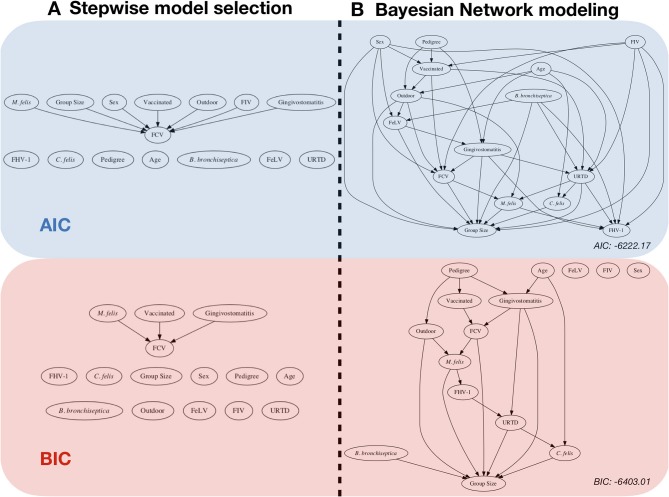
Risk factor analysis with the FCV dataset. Comparison between: **(A)** classical stepwise multivariable model selection using two different model scores, represented by a network (backward-forward model selection algorithm), and **(B)** Bayesian network (BN) analysis with two scores and the corresponding network (using an Additive Bayesian Network algorithm). For ease of comparison, we chose to present the classical regression approach in a network format as well. The blue part was performed by using the AIC (Akaike Information criteria) score, and the red part was performed by using the BIC (Bayesian Information Criteria) score. Stepwise model selection and BN modeling using the same score gives very similar results. The BN models depict a more detailed description of the data. The objective of this paper is to describe how to obtain and to interpret such a result.

Classical regression is the most popular method in epidemiology for performing risk factor analysis (see Figure 1). Regression analysis is a powerful, robust, and versatile statistical approach that estimates the relationship between two or more variables of interest. There are many types of regression analyses. At their core, they all examine the influence of one or more independent variables or factors on a dependent variable (also called the outcome or exposure variable) (3). The epidemiologist's expert decision about which variable is the response drives the regression. The philosophy for performing a risk factor analysis is to use a significance metric to extract relevant (influential) factors. However, this approach becomes unstable when the level of collinearity is too pronounced within the factors. In this context, collinearity means that some factors are (to a certain degree) predicted by a set of others, since the dataset contains redundant information. It is possible to identify and to remove redundancies, but the instability could remain due to inherent correlations in the system being studied. For identifying collinearity, some techniques have been proposed: e.g., changing estimated regression coefficients when a predictor variable is added or deleted, calculating the variance inflation factor (VIF), and deleting factors with large VIF. Some tests have been proposed, but no consensus exists on their usefulness. A statistically related problem arises when sub-selecting a limited number of variables, either due to redundancy within the data or due to computational limitations. A well-adapted approach to multivariate system epidemiology is the so-called Minimum Redundancy Maximum Relevance model (4). In the presence of collinearity, regression analysis is known to become unstable and to predict the effect of individual factors poorly. A high correlation among factors is common in epidemiology when studying biosecurity [e.g., (5)]. In this context, a direct association means that the set of variables will change the outcome when their values change. An indirect association is a correlation mediated by an intermediate set of variables. In epidemiology, it is important to model and identify variables that have a direct impact on the variable of interest. The directly associated variables are primary targets for intervention or for identifying the best candidate for knowledge-seeking from a modeling perspective. This process is called “structure discovery” in machine learning (6). The easy-to-interpret quantitative outputs and the holistic qualitative outputs of a typical BN model make it useful in observational analysis and a good alternative to classical methods.

Other popular graphical machine learning techniques exist, such as artificial neural networks (ANNs), regression trees, or random forest. At the outset, BN, ANN, and tree analyses look alike, as they rely on directional graphs. However, they are different approaches and should not be confused. We will not discuss the particular case of causal Bayesian networks in this paper because causal inference requires theoretical assumptions that are beyond the scope of this paper, and the methodology can become very field-specific. We refer the interested reader to Pearl and Mackenzie (7) for an extensive overview of modern causal modeling using graphical models. The general task addressed in this paper is, from an observational dataset, to find a suitable network that represents the relationships between the variables well using probabilistic methods. This paper seeks associations rather than causal links, i.e., exploratory analysis rather than confirmatory analysis.

This paper is structured as follows. A motivating example is presented in section 1.1. Section 2 gives a brief overview of the basic principles and an overview of the use of BNs in other fields. Section 3 gives a detailed presentation of the BN methodology, including a discussion of the key terms relevant to the BN modeling landscape. It also outlines some rules for good modeling. Section 4 lists the main commercial and non-commercial software implementations. Section 5 presents a case study with the FCV dataset. Finally, section 6 discusses the limits and misuse of BN models in epidemiology.

### 1.1. Motivating Example

**Figure 2:**
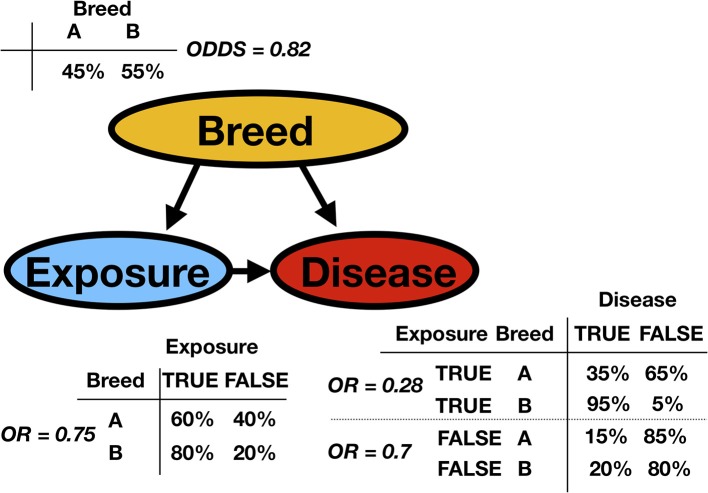
A simple synthetic discrete Bayesian network is presented (represented as a DAG) with the conditional probability tables (CPT) and the corresponding odds or odds ratios (OR). A certain disease is studied in animal production. The observed variables are the animal's breed and the exposure to sick animals. In the DAG, *Breed* is the parent of both *Exposure* and *Disease*. *Exposure* is the parent of *Disease*, as is indicated by the arrow. Inversely, one can say that *Disease* is the child of *Exposure*. From the column or row sums of the CPTs, it is possible to extract the marginal probabilities.

Consider the fictitious example of an observational study about a particular disease in animal production. In the population, there are two breeds. The exposure status for each animal, the breed variable, and the disease status have been recorded (Possible values for exposure status are true or false and represent, for example, contact with sick animals. Possible disease status values are true or false). Based on this observed dataset, the task is to analyze the data. Figure 2 presents the network (the qualitative part of the model) with the so-called conditional probability tables (CPTs, the quantitative parts of the model). The CPTs are, in this discrete case and for mutually dependent variables, matrices displaying the conditional probability of a given variable with respect to the other. The classical approach would perform a regression analysis with a disease as the response and exposure and breed as factors. It would create an essentially one-dimensional BN because it would overlook the existing link between exposure and breed. We would ignore the fact that breed B is much more likely to be exposed than breed A. In epidemiology, this is a possible confounder. Indeed, breed is associated with both the dependent variable and independent variable, possibly causing a spurious association between exposure and disease status. To be classified as a confounder, breed must be causally related to exposure and disease, with no link between exposure and disease beyond the confounding effect (8). Many methods have been proposed to control for confounders in observational studies: stratification, restriction, matching, propensity score adjustment, and multiple BN regression models.

A related but different issue is the so-called effect modifier or interaction phenomenon. Interactions may arise when considering three or more variables when the effect of one variable on an outcome depends on the state of a second variable: in other words, when the effects of the two causes are not additive. An interaction can also be described as an acausal association. A typical example among humans is the interaction that exists between ultraviolet light (UV) and analogs of vitamin D (VitD) or its precursors in bone metabolism. As Lebwohl et al. (9) show, with an insufficient amount of UV light, VitD (or its precursors) will not affect bone metabolism. Symmetrically, UV light without VitD (or its precursors) will not affect bone metabolism. BN modeling is conceptually attractive for performing this task in analyzing the variables within a network. From a mathematical perspective, the global model (i.e., the network and model parameter) is called the joint probability distribution.

## 2. Background and Objectives

A BN modeling approach was proposed more than 30 years ago (10). It has a track record of successful applications using real-world data in a wide variety of domains. BNs are used for modeling beliefs in social sciences (11), decision support (12), biology (13), and finance and marketing (14). More recently, this approach has been applied in veterinary epidemiology (15, 16), anti-microbial resistance (17–19), and animal welfare (2).

As BN models are used in a wide variety of research fields, they are called many different names. Here is a (non-exhaustive) list of terminology: Bayesian networks, belief networks, decision networks, probabilistic directed acyclic graphical models, recursive graphical models, naive Bayes, causal probabilistic networks, or influence diagrams (20).

Fitting BN networks to data is called *learning*. This term comes from the machine learning community and is a synonym for selecting the best network. Learning a BN from a dataset entails estimating the joint probability distribution, which encodes the global probability distribution of a multi-variable problem. When multiple variables are mutually dependent, calculating the joint probability distribution is useful as one could compute two other distributions: the marginal distribution, giving the probabilities for any variables independently of the other variables, and the conditional probability distribution, giving the probabilities for any subset of the variables conditional on particular values of the remaining variables. It is usually a two-step process involving (i) structure learning and (ii) parameter learning. This is globally called the *structure discovery* process (6). The next section presents a detailed overview of these methods. Once the joint probability distribution is estimated, it can be graphically represented using a Directed Acyclic Graph (DAG), i.e., a Bayesian Network. A BN is essentially a visual representation of a probabilistic model.

## 3. General Methodology

We now present the general methodology and the steps needed to fit a BN model to data. First, a short introduction to Bayesian networks is given. Then, a description of the two main learning classes of algorithm is given. Afterward, the Additive Bayesian Network (ABN) methodology is presented in detail as a special case of BN modeling. A closely related methodology is Structural Equation Modeling (SEM) (21). SEM includes different methodologies, such as confirmatory factor analysis, path analysis, partial least squares path modeling, and latent growth modeling. Although they share the same purpose, SEM and BN methodologies have significant differences (22). SEM uses a causal approach based on cause-and-effect thinking, whereas BN is based on a probabilistic approach. SEM is well-suited to latent variable modeling (i.e., variables that are not directly observed but are modeled from others), which is not possible in the BN methodology. This is often the primary motivation for using SEM. A BN model can take advantage of new data, whereas SEM cannot.

### 3.1. Bayesian Network

In a BN model context, a statistical model represents the data-generating process encoded using a graph and the parameter estimates. It can be used to describe the data, generate knowledge (i.e., understanding), or make predictions. The BN graphical representation consists of nodes, which are the random variables, and edges, which form the relationships between them. These representations often use odds ratios for discrete variables and correlation coefficients for continuous variables. The network structure should be directed and contain no cycles.

**Figure 3:**
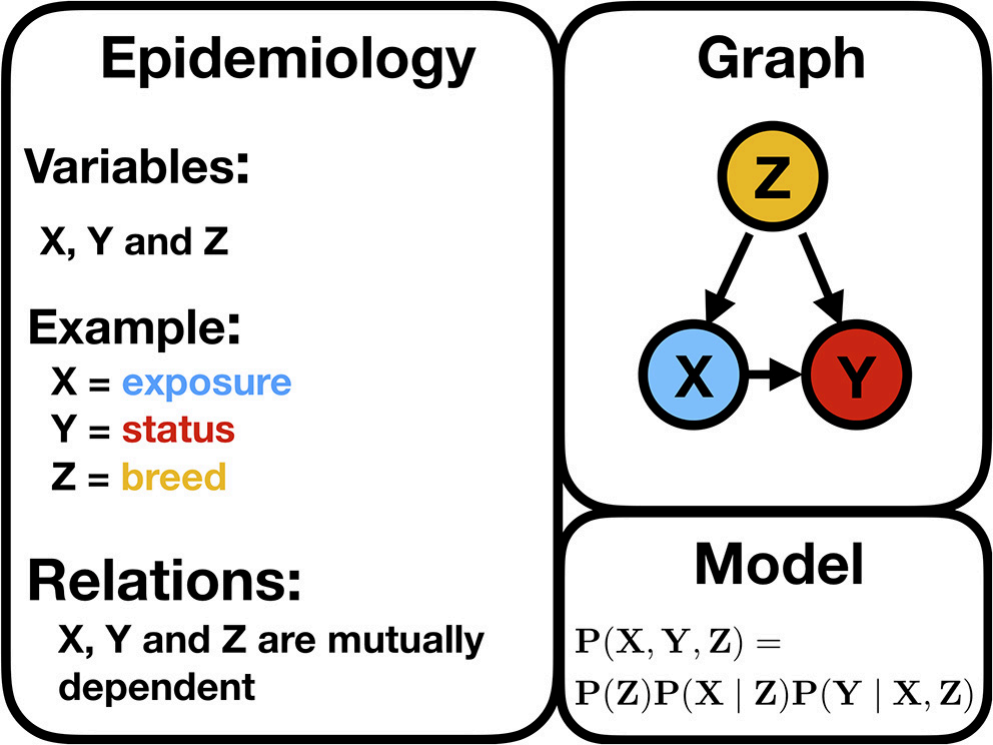
Illustration of a typical confounding situation described in the text stating the biologically expected relationships, a DAG, and the corresponding statistical model encoding the joint probability distribution.

Figure 3 illustrates a typical confounder situation. In veterinary epidemiology, *X* could be exposure, an intervention, or a certain condition; *Y* is the animal status; *Z* is a confounder, such as sex, breed, age, or body mass index (BMI). Figure 3 is a BN, and the following formula gives its encoded probabilistic model, indicating how to encode the joint probability distribution [i.e., *P*(*X, Y, Z*)] into a product of the conditional distributions

(1) $$\begin{array}{l} P\left(X,Y,Z\right)=P\left(Z\right)P\left(X|Z\right)P\left(Y|X,Z\right), \end{array}$$

where *P*(. ∣ .) stands for the conditional probability distribution. In a discrete setting, this probability is given by the CPTs. Hence, *P*(*X*∣*Z*) is the conditional probability of an animal of *breed* = 1 being exposed. *P*(*exposure* = *TRUE* ∣ *breed* = 1). Based on the CPT, the odds ratio could be computed as the cross product of entries of the contingency table. The general formula to deduce the probabilistic model from a BN implies that the joint probability [here *P*(*X, Y, Z*)] factorizes as a product of the conditional probabilities of the variables, given their set of parents

(2) $$\begin{array}{l} P\left(\text{X}\right)=\overset{n}{\underset{j=1}{\prod }}P\left(X_{j}|{\text{P}\text{a}}_{j}\right), \end{array}$$

where **X** is the set of random variables (i.e., the dataset), *X*_*j*_ is the *j*th random variable, and **Pa**_*j*_ is the set of parents of the *j*th random variable. From a mathematical point of view, a DAG is a union of two sets: the set of nodes and the set of arrows. The network structure has a probabilistic interpretation. It encodes the factorization of the joint probability distribution of the dataset. One significant consequence of the duality between probabilistic models and network structures is that multiple different graphs can represent a given probabilistic model. As shown in Supplementary Material, only specific arrangements matter when selecting networks. The exact structure of a BN is not unique, and so interpretation of the effect of a variable based on arc direction, e.g., as in a typical causal statement that variable *X* impacts *Y*, is generally not valid. Therefore, on the other hand, caution is needed to avoid overinterpreting the results of a BN graph. Removing all arcs and presenting only undirected networks may potentially remove some useful information.

### 3.2. Model Learning

In a Bayesian setting, the model's posterior distribution given the data factorizes as the product of the structure's distribution given the data and the model parameters given the structure and the data. Then, the learning phase is formalized as:

(3) $$\begin{array}{l} P\left(M|D\right)=\underset{\text{model learning}}{\underset{︸}{P\left(\theta_{M},S|D\right)}}=\underset{\text{parameter learning}}{\underset{︸}{P\left(\theta_{M}|S,D\right)}}\cdot \underset{\text{structure learning}}{\underset{︸}{P\left(S|D\right),}} \end{array}$$

where M is the full model (i.e., the network structure S and the parameter estimates $\theta_{M}$) and D is the dataset. One can see from Equation (3) that the two learning steps are intertwined and mutually dependent.

Learning BNs from a dataset is very complicated from the programming and statistical perspectives because the number of possible models is massive. For example, the total number of possible DAGs with 25 nodes is larger than the number of atoms in the universe (10^80^), so the number of possible networks grows faster than the exponential function, i.e., super-exponentially. Thus, it implies the use of a smart and efficient algorithm and controlling for possible overfitting (23). In situations with limited data and numerous models, any selection or constructive method risks producing overly complicated models (i.e., a network with too many arcs) to represent the data. A key feature of the described methods is the ability to control for overfitting and so produce parsimonious models.

### 3.3. Structure Learning

In order to select BNs from observed data, two main approaches have been proposed: *constraint-based* and *score-based* approaches. These approaches, which are based on different statistical paradigms, are typically performed in a semi-supervised setting. Despite the intention of selecting structures in a fully data-driven way, it is possible to guide the learning phase with external knowledge. A fully-supervised approach entails asking experts to design a network and fit it to the data. In practice, this is often not possible due to the high number of possible models that make this task highly complex. However, a semi-supervised method could be suitable, as typically, partial previous knowledge exists on specific research topics. The set of assumptions under which the learning algorithms are working are: each node in the network is a random variable (i.e., not a function of the other variables), the relationships between the random variables should be modeled with conditional independencies, every possible combination of the random variables should be plausible (even if very improbable), and the data should be derived from independent realizations of an unknown model (without temporal or spatial dependencies) (6).

#### 3.3.1. Constraint-Based Algorithm

Constraint-based algorithms take advantage of the significant differences between colliding arrows (v-structure) and other types of structures in BN. Multiple methods exist, but one popular procedure identifies the set of mutually possible dependent variables to reduce computational complexity. It constructs the skeleton of the graph by searching for which variables are or are not related, regardless of the arc's direction. Finally, conditional independence tests are performed to detect v-structures (24). They are used as an oracle to decide on the inclusion or exclusion of an arc between two variables. Finally, based on the skeleton and the v-structure, the procedure generates (partly) directed graphs. This approach was proposed by Verma and Pearl (25). Since then, many refinements have followed. It is the methodology of choice for performing causal inference. It is also known to be more efficient with sparse networks, i.e., with a limited number of expected arcs.

Choosing the independence test framework, i.e., both the algorithm and the tests themselves, is this approach's major limitation. Another subjective user choice is how to set the significance level of the tests (classically called in statistics the α level). This choice is known to be field- and data-specific, and it influences the learned network (26). If the number of tests is substantial, precautions must be taken to avoid the problem of multiplicity, such as using Bonferroni's correction factor. Another drawback of this approach is the fact that it produces only one model.

We next present a methodology that can produce a family of plausible networks. They could be mixed to generate a more robust network. When data are scarce, it is reasonable to believe that multiple competitive models could be identified with a high level of confidence, and it would be hard to select only one.

#### 3.3.2. Score-Based Algorithm

Bayesian network modeling can be viewed as a model selection problem. The most popular approach to BN modeling scores the candidate model in a stepwise procedure and selects the model that has the optimal score. The most popular implementation is based on an AIC (Akaike information criterion) (see Figure 1). The paradigm used here ensures that, if the score is well-designed, the selected model (i.e., the one with the optimal score) should represent the data well. Many scores dedicated to BN have been proposed depending on the nature of the data (for example, whether they are discrete, continuous, or a mixture of different data distributions). These scores have been designed to be penalized for model complexity because, according to Occam's razor principle, if two models for a given phenomenon exist, then the simpler should always be preferred.

To select the optimal network, i.e., the network that optimizes the network score, one needs to have a search algorithm. In contrast to the learning phase, this search algorithm only aims at finding the network with the highest possible score. Multiple algorithms have been proposed in the literature. One can perform a so-called *exact* search or use a *heuristic* approach. The *exact* search is only possible on a desktop computer for a very limited network size with a maximum of 20 nodes. Heuristic search algorithms, however, leading to an approximately optimal network, scale well with the network size (number of nodes in the network).

A score-based algorithm can learn the conditional independence between variables, so an entirely directed network could generally lead to an acausal interpretation of the arrow's direction. Thus, a score-based algorithm encodes statistical dependencies and not causal links. As Pearl (27) states:

It seems that if conditional independence judgments are by-products of stored causal relationships, then tapping and representing those relationships directly would be a more natural and more reliable way of expressing what we know or believe about the world. This is indeed the philosophy behind causal Bayesian networks.

In a score-based perspective, arrows are important and could be displayed even if their interpretation is not fully causal. Some authors still advise that the skeleton of the network be displayed; this is also a valid approach and depends on the nature of the problem studied (28).

The major limitation of the score-based approach is the score used. Indeed, a well-designed score should minimally differentiate structures with different probabilistic models (as shown in **Figure 6**). A lot of theoretical effort has been put into deriving likelihood equivalent scores (i.e., score differentiate equivalence classes of BN), which have only been accomplished under very restrictive assumptions. For example, scores that preserve likelihood equivalence with a general mixture of data distributions do not exist. The classical workaround is to discretize the data, and then suitable scores exist. It is known in epidemiology that discretization, though common, has severe consequences and is not always advisable (29). Finally, it is interesting to note that when the number of observations is large enough, the constraint-based and score-based approaches are equivalent, and there is usually no particular reason for choosing one over the other.

### 3.4. Parameter Learning

Once the network structure has been selected, parameter learning can be performed locally. Only the local structure is required: the index node and the set of parent nodes. Two main approaches exist for estimating the parameter distribution: the *maximum likelihood* and the *Bayesian* approaches. The choice of the structure learning algorithm does not influence the method for learning the parameters of the BN. Those two methods are based on two different statistical frameworks. The *maximum likelihood* assumes an unknown but fixed set of parameters for maximizing the likelihood, whereas the *Bayesian* approach treats the parameters as random and assumes a prior to them. They are computed from the posterior distribution of the parameter of the network. The main consequence is linked to the prior's choice. Indeed, the prior can help to estimate the parameters when there is not enough information within the data (30). The model parameters are interpretable as regression coefficients. Those parameters are central in model interpretation, as they give the direction of the effect and the effect size.

In the presence of missing data, more sophisticated techniques should be used to infer model parameters. In a BN context, the missing data mechanism should be ignorable (31), i.e., the data should be Missing at Random (MAR) or Missing Completely at Random (MCAR). Indeed, the MAR assumption is the minimal condition on which statistical analysis can be performed without modeling the missing data mechanism. The most popular approach for computing the value of the likelihood of the dataset with incomplete data is the Expectation-Maximization (EM) algorithm (32, 33). This is an iterative procedure for estimating the maximum *a posteriori* of a statistical model in two steps (E-step and M-step). An alternative method is variational inference, which provides a computationally cost-effective lower bound on the marginal likelihood (34). Hybrid algorithms are possible. When such solutions are not available, the usual workaround is to perform a complete case analysis, i.e., ignoring all observations containing missing information. The obvious disadvantage is the loss of existing knowledge. As an alternative, a model imputation strategy allows the researcher to still use the existing information and create data when they are missing. A good quality check is to perform both analysis and testing if they give fundamentally different results.

### 3.5. Additive Bayesian Network

The Additive Bayesian Network (ABN) methodology is a score-based methodology that takes advantage of a particular model parametrization. It uses the robustness and the full range of applicability of the regression framework to parameterize the network. It is used to score the candidate network and to estimate the model's parameters. The regression framework could be set in a Bayesian or a frequentist setting. The regression coefficient estimates in both Bayesian and frequentist settings are usually close to one another, but the network scores could be different, creating a very different network for each setting. Indeed, in a Bayesian setting, the so-called marginal posterior network score distribution is returned, whereas in a frequentist setting, one of the typical model selection scores is used [the Akaike Information Criterion (AIC), Bayesian Information Criterion (BIC), or Minimum Distance Length (MDL)]. The term BN might be misleading, as BN models do not necessarily imply a commitment to Bayesian statistics. From a formal perspective, ABN takes advantage of the exponential family to parameterize the model and to enable the mixing of different kinds of data, such as continuous, discrete, or Poisson-distributed. A nice by-product of this parametrization is that it also allows a user to measure uncertainties of the model parameters. In a *Bayesian* setting, the credibility intervals can be computed, whereas in a *frequentist* setting, the confidence intervals can be computed. The term *additive* in ABN refers to the assumption that the effects of the variables are additive.

**Figure 4:**
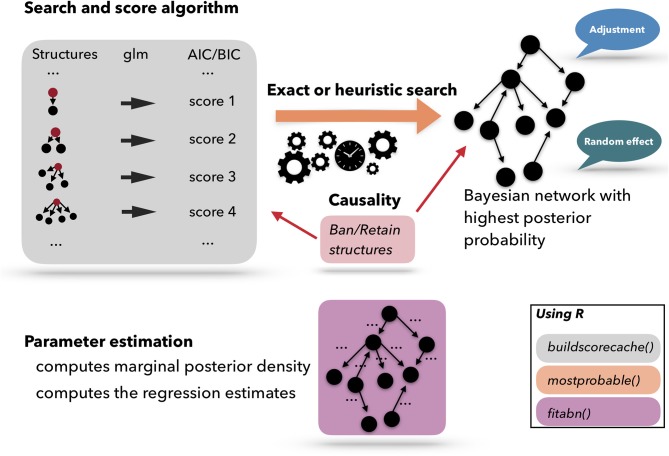
Schematic representation of the Additive Bayesian Network workflow. First, a cache of scores is computed for atomic networks. Then, a search algorithm is used to optimize the comprehensive network. Based on the optimized network, the model parameters are learned. Those three steps are programmed in three different R functions: buildscorecache(), mostprobable(), and fitabn().

Figure 4 presents a scheme of the workflow used for performing an ABN analysis. A list of pre-computed scores based on atomic networks is calculated. The atomic networks are a given node with all possible combinations of parents. The list of atomic networks with their scores is called the cache of scores. Based on the cache of the pre-computed scores, a search algorithm is used to optimize the network. The search algorithm can be heuristic or exact. Based on the optimized network, the model coefficients can be fitted. ABN is thus, essentially, a graphical modeling technique that extends the usual Generalized Linear Model (GLM) to multiple dependent variables through the factorization of their joint probability distribution. In epidemiology, data are commonly generated in a setting that has an apparent grouping aspect, for example if the data are collected in different countries, counties, farms, etc. In statistics, it is known that clustering, due to the potential non-independence between data points from the same cluster, could cause over-dispersion. One of the many advantages of using the ABN framework is that it allows for adjustments for clustering within the Bayesian setting. ABN and other score-based approaches have the feature of letting the user impose external causal inputs (such as banning or retaining arcs based on previous scientific knowledge) to ensure the model's interpretability. Additionally, to considerably simplify the search space, the degenerescence between different equivalent DAGs, i.e., different networks sharing the same score, can be lifted.

ABN relies on priors at different levels. In the structure learning phase, one needs to decide on a structural prior, which encodes how likely a given structure is. In ABN, a form of prior is used that assumes that the prior probabilities for a set of parents comprising the same number of parents are all equal. It favors parents sets with either a very low or very high number of parents, which may not be appropriate. Alternatively, an uninformative prior is used where parent combinations of all cardinalities are equally likely. When using the Bayesian implementation during the model parameters learning phase, priors are used for estimation. Those priors are designed to be uninformative.

Any BN modeling approach contains approximations to make the process computationally tractable. The most common approximation is to limit the number of possible parents per node, i.e., the model complexity. Another approximation is linked to the nature of epidemiological data. Multiple types of distributions often co-exist within a dataset, and the score used should be versatile enough to handle them. From a mathematical perspective, this leads to an approximation. As previously mentioned, the chosen search algorithm could also imply some approximations. Thus, the global ABN method relies on multiple approximations, and the end-user should be aware of them. Transparently reporting them is of paramount importance.

## 4. Software Implementations

Many commercial and non-commercial implementations for BN modeling techniques exist. Commonly commercially used BN software includes the Hugin Decision Engine produced by Hugin Expert (www.hugin.com) (35), and there is an R package to interface the Hugin Decision Engine with R: RH ugin. Further examples are bayesfusion (www.bayesfusion.com), netica (www.norsys.com), and BayesiaLab (36). A popular implementation tool in MATLAB is the Bayes Net Toolbox (BNT) (37).

Within the epidemiology community, a popular open-source programming language is R (38). There are multiple R packages targeting BN modeling. The bnlearn R package contains many score-based and constraint-based algorithms, as well as multiple searching procedures (39). It is the largest and probably the most popular R package for BN modeling. When targeting causal BN inference, the pcalg R package is the most used package (40). It has a unique implementation of the PC-algorithm. The catnet R package deals with categorical data only (41). The deal R package handles both continuous and discrete variables (42). It is one of the oldest R packages for structure and parameter learning. From a more general perspective, the gRain R package is designed to perform inference in probabilistic expert systems where BNs are a special case (43). The abn R package has an implementation of a score-based system in a Bayesian and in a frequentist framework. It also has a unique implementation of an exact search algorithm and targets mixed-distributed datasets. The supported distributions are *multinomial, Bernoulli, Gaussian*, and *Poisson*. The abn R package can deal with random effects for controlling possible clustering within the data. All those packages are distributed via CRAN. Task View: gRaphical Models in R (CRAN.R-project.org/view=gR) gives a very comprehensive overview of the different computing packages available on CRAN.

## 5. Case Study

For a case study, we focus on the Feline calicivirus (FCV) infection among cats in Switzerland. FCV is a virus that occurs worldwide in domestic cats but also in exotic felids. FCV is a highly contagious virus that is the major cause of upper respiratory tract disease or cat flu in felids. This is a disease complex caused by different viral and bacterial pathogens, i.e., FCV, Feline Herpes Virus 1 (FHV-1), *Mycoplasma felis (M. felis), Chlamydia felis (C. felis)*, and *Bordetella bronchiseptica (B. bronchoseptica)*. It can be aggravated by retrovirus infections, such as Feline Leukemia Virus (FeLV) and Feline Immunodeficiency Virus (FIV). This composite dynamic makes it very interesting for a BN modeling approach.

The data were collected between September 2012 and April 2013. Berger et al. (44) presented the original data and analysis and investigated the frequency of FCV in cats with FCV-related symptoms and in healthy cats in Switzerland. They also investigated potential protective and risk factors. The FCV dataset includes multiple viral and bacterial pathogens, retrovirus, clinical signs, and animal-related risk factors. The potential risks or protective factors are expected to be interrelated and correlated. The FCV dataset entries are described in Table 1. The variable sex is a composite variable between a cat's sex and reproductive status with four possible values: male, male neutered, female, female spayed.

**Table 1:** Description of the factors in the FCV dataset.

**Variable's name**	**Description**
FCV	Feline calicivirus status (0/1)
FHV-1	Feline herpesvirus 1 status (0/1)
*C. felis*	*Chlamydia felis* status (0/1)
*M. felis*	*Mycoplasma felis* status (0/1)
*B. bronchiseptica*	*Bordetella bronchispetica* status (0/1)
FeLV	Feline leukemia virus status (0/1)
FIV	Feline immunodeficiency virus status (0/1)
Gingivostomatitis	Gingivostomatitis complex status (0/1)
URTD	Upper respiratory tract disease complex (0/1)
Vaccinated	Vaccination status (0/1)
Pedigree	Pedigree (0/1)
Outdoor	Outdoor access (0/1)
Sex	Sex and reproductive status (male, male neutered, female, female spayed)
Group size	Number of cats in the group-housing (count)
Age	Age in years (continuous)

*The variable names used in R are slightly different than the ones used in the text and the figures*.

The FCV dataset is a good candidate for a BN analysis, as complex and intertwined relations are expected among multiple recorded viruses and bacterial pathogens, animal-related variables, and environmental contributions. A major difference between this case study and the original study is that the original study design included two groups of cats: those in which FCV infection had been suspected (based on clinical signs) and healthy cats, as determined by a veterinary practitioner based on an unremarkable physical examination. The present analysis discards this study characteristic and analyzes the data as a whole observational dataset. This might hamper comparability with the original analysis so that the prevalence would not be estimable anymore.

The study enrolled 300 cats, i.e., the healthy and the FCV-suspected cats as a unique observational group. A subset of 20 of the 300 observations contain missing values. As the ABN approach requires a complete case dataset, a model imputation approach, using random forest, was used to fill in the missing data (45). Missing data are a common problem in veterinary epidemiology, and no single solution exists. However, as general advice, one can perform a complete case analysis and an imputed one. If the findings are similar, this is a good indication that there is enough information in the data to estimate an ABN model. If the findings differ significantly, then more investigations should be conducted to model the missing data. The dataset is made of 15 variables: one of them is continuous, one of them is integer-distributed, and the others are discrete.

**Figure 5:**
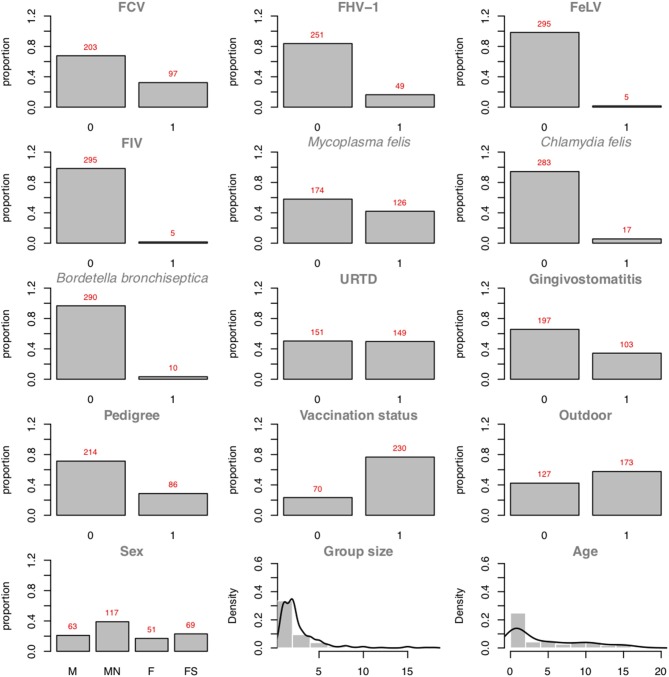
Plots of the variable distributions in the FCV dataset. Discrete data are represented using their proportions. Continuous data are represented using histograms and their densities (*Group Size* and *Age*).

Figure 5 presents the plots of the distributions of the individual variables. As one can see, 97 positive cases among the 300 cats are recorded. In binary logistic regressions, a popular factor of performance is the ratio between the smaller number of the two-outcome group (i.e., number of events) divided by the number of regression coefficients (excluding the intercept). In the FCV dataset, the Event Per Variable (EPV) is 97 cases divided by 7 variables (the maximum number of parents allowed), which equals 13.86 (for the *outcome: FCV*). van Smeden et al. (46) suggested that low EPV has a smaller impact than data separation or total sample size. The abn R package comes with a workaround dedicated to specifically managing low EPV and data separation: Firth's correction. The data separation problem occurs in logistic regression models when a certain combination of factors contains no observations. For example, in the FCV dataset, no record of a male cat with an FCV-positive status would imply that the sex of the cat perfectly predicts the FCV status, and the regression estimates would become numerically unstable. Firth's correction aims at producing reliable estimates in (quasi-)separated datasets. As abn tests all possible combinations of variables, the risk of data separation, especially in small datasets, is high.

### 5.1. Additive Bayesian Network Analysis

An ABN analysis is performed with the sequential computation of three functions: buildscorecache() (pre-computed scores), mostprobable() (a search algorithm), and fitabn() (parameter learning). At this stage, the user should perform multiple steps before starting an ABN analysis: loading and formatting the data, setting up the distribution of each network's nodes, deciding on possible prior knowledge, and deciding on the maximum number of parents per node (i.e., limiting the network complexity).

### 5.2. Loading the Data

The FCV dataset is accessible through the abn R package:

R> data("FCV", package = "abn")

### 5.3. Setting Up the Distributions List

The user should define a list of distributions to let abn know how to fit the data. This is similar to the family statement in the R function: glm(…, family = binomial(link = “logit”), …). One needs to create a named list that contains all the variable names and the corresponding distributions. The available distributions are *binomial, Gaussian, Poisson*, and *multinomial*, where the last distribution is available with MLE scores only.

R> mydists  <- list(FCV = "binomial",
+ FHV1 = "binomial",
+ FeLV = "binomial",
+ FIV = "binomial",
+ Mfelis = "binomial",
+ Cfelis = "binomial",
+ Bbronchiseptica = "binomial",
+ URTD = "binomial",
+ Gingivostomatitis = "binomial",
+ Pedigree = "binomial",
+ Vaccinated = "binomial"1,
+ Outdoor = "binomial",
+ Sex = "multinomial",
+ GroupSize = "poisson",
+ Age = "gaussian")


Binomial and multinomial data should be coerced to factors, and Gaussian and Poisson should be treated as numeric. As the list of distributions contains a *multinomial* node, the MLE method will be used in this case study.

### 5.4. Prior Knowledge

The prior knowledge in the abn R package can be defined by using two different means: a matrix or formula-wise statements. In the FCV dataset, the three factors *Sex, Age*, and *Pedigree* should not have a parent node. In other words, those factors cannot be influenced by any other variables within the dataset, and this prior knowledge should be transferred to abn to ensure the biological plausibility and interpretability of the final model. In practice, this is done by banning or retaining arcs within the network. By default, abn assumes no banned or retained arcs. See ?fitabn in R about how to specify banned or retained arcs by using a formula-like syntax.

### 5.5. Parent Limit

To define the number of parents per node needed, one usually performs an ABN analysis in a *for loop* and increases the number of parents at each run. One computes the network score at each run and stores it. The number of parents needed is the number which leads to an unchanged network score. The code displayed below performs the so-called *parent search* for AIC, BIC and MDL scores:


 
  R> aic.values  <- aic.values  <- mdl.values  <- vector(length = 11)
  R>
  R> #for loop to discover the suitable network complexity
  R> for (i in 1:11) {
  + max.par  <- i
  + # construction of the score cache
  + mycache  <- buildscorecache(data.df = mydata,
  + data.dists = dists,
  + dag.banned =  ∼Sex|.+Age|.+Pedigree|.,
  + max.parents = max.par, method = "mle")
  + # optimal dag with BIC
  + dag  <- mostprobable(score.cache = mycache, score = "bic")
  + fabn  <- fitabn(object = dag, method = "mle")
  + bic.values[i]  <- fabn$bic
  + # optimal dag with AIC
  + dag  <- mostprobable(score.cache = mycache, score = "aic")
  + fabn  <- fitabn(object = dag, method = "mle")
  + aic.values[i]  <- fabn$aic
  + # optimal dag with MDL
  + dag  <- mostprobable(score.cache = mycache, score = "mdl")
  + fabn  <- fitabn(object = dag, method = "mle")
  + mdl.values[i]  <- fabn$mdl
  + }


**Figure 6:**
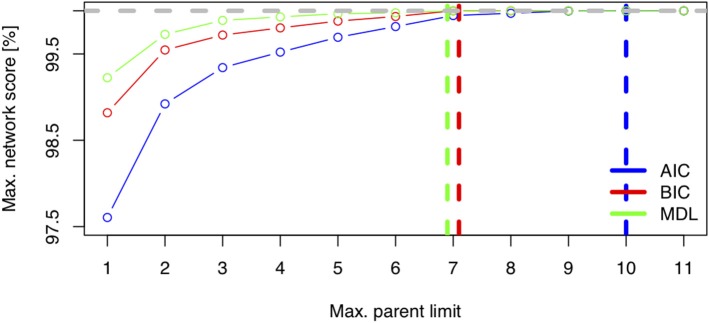
Network score: Minimum Distance Length (MDL), Bayesian Information Criteria (BIC), and Akaike Information Criteria (AIC) as a function of network complexity, i.e., the maximum number of allowed parents per node.

Figure 6 displays the network score achieved in percent of the absolute maximum as a function of the maximum allowed number of parents per node for three scores (AIC, BIC, MDL). The maximum needed number of parents per node depends heavily on the chosen score. The AIC's learned network requires ten parents per node, whereas the BIC's learned network and the MDL score require only seven parents per node. This is coherent with Figure 1, where both stepwise model selection and BN modeling approaches with AIC select a more dense network than with BIC. Thus, choosing the score is an important modeling decision. For this case study, the BIC score will be preferred. The rationale for this subjective modeling choice is the following: since BIC is more parsimonious in terms of model complexity (considering the number of possible relationships within the network) with a limited number of observations, it is very popular with BN analysis and is closer to a Bayesian score. Based on that information, an *exact search* can be performed using the mostprobable() function. In the eventuality that the number of nodes would exceed 20, we would have to rely on a heuristic approach. The function searchHillclimber(), for example, performs multiple greedy hill-climbing searches and returns a consensus network based on a user-defined thresholding percentage. It is a good alternative when an exact search is not possible for computational reasons.

### 5.6. Control for Robustness and Accounting for Uncertainty

The next and final modeling step is 2-fold. It aims to control for overfitting and to account for uncertainty in the model. In statistics, and more generally in data analysis, overfitting is the production of an analysis that too closely represents the data and thus may poorly generalize findings. Overfitting produces an overly complicated model that captures unnecessary features of the studied problem. Underfitting produces a model that is too simple and thus does not capture an essential features of the studied problem. Both under- and over-fitting are limiting factors for the reliability of any analysis, but in BN modeling, the risk of overfitting is known to be high, so measures should be taken for controlling it. Multiple approaches have been proposed to manage the tendency of BN modeling to overfit the data. Parametric (2) and non-parametric (16) bootstrapping are very popular methods. In this case study, we use a structural Monte-Carlo Markov Chain (MCMC) sampler implemented in the mcmcabn R package (47). This approach allows us to construct MCMC samples respecting global structural priors and the chosen constraints (see Supplementary Material for details). The output of this modeling step estimates the probabilities of each arc's existence in the DAG. Based on those probabilities, the final DAG is pruned by removing the arcs that do not have enough support. Additionally, structural MCMC can be computationally faster for a certain class of problems.

As a by-product, the Bayesian model averaging approach helps to account for uncertainty during the modeling process. It is plausible to imagine that multiple DAGs are realistic for the data and that the limited sample size of the FCV dataset does not let us select among them objectively. Bayesian model averaging is a technique for reporting the acceptable manifold models. It has shown promising and impressive results with real-world data in closely related research fields (48–50).

From an applied perspective, so-called *structural queries* are very attractive features deducible from the MCMC sample. Structural queries are typical questions the researchers can ask the models, such as: *What is the probability that the classical signs of URTD (nasal discharge, ocular discharge, conjunctivitis, and sneezing) are NOT associated with the FCV status?* (99.7%); or *What is the probability of the gingivostomatitis complex being directly associated with the FCV status if the vaccination status is NOT?* (50%). These modeling queries are typically laborious to address with classical statistical methods, making Bayesian model averaging a very promising complementary approach to BN modeling.

### 5.7. Presentation of ABN Results

**Figure 7:**
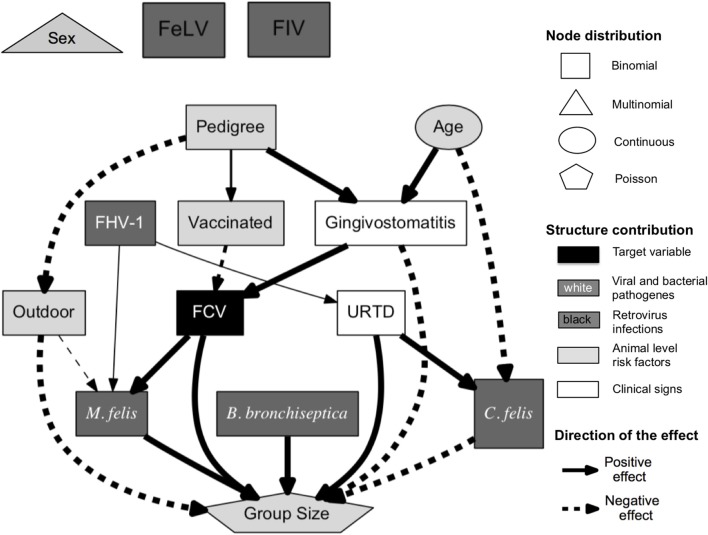
Pruned additive Bayesian network model selected using BIC score, with a maximal parent limit of seven, constructed by pooling results across 100,000 MCMC moves [thinning factor of 100, burn-in phase of 17% of the total number of steps, and non-informative global network prior (except the already mentioned network restriction)]. The squares are binomial, the pentagon is Poisson, ovals are Gaussian, and the triangle is based on multinomial distributed variables. The FCV node is directly associated with the factors vaccination status, gingivostomatitis, *M. felis*, and group size. The solid arrows represent positive effects and the dashed arrows, negative effects. The thickness of the arrow is proportional to the support probability. The gray scale values of the nodes encode their level of contribution (target variable, viral and bacterial pathogens, retrovirus infections, clinical signs, and animal level risk factors).

An ABN analysis produces qualitative results (see Figure 7) and quantitative results (see Table 2). Figure 7 displays a pruned additive Bayesian network model constructed by pooling results across 100,000 MCMC moves. A thinning factor of 100, a burn-in phase of 17% of the total number of MCMC steps, a non-informative global network prior, and a thresholding factor of 50% were used. The original DAG was obtained using the BIC network score and seven parents per node at maximum. Three prior knowledge constraints were incorporated into the analysis: the nodes *Sex, Age*, and *Pedigree* cannot have any parents. The original DAG (presented in Figure 1 in the red square on the right) has 20 arcs. The pruned one (see Figure 7) has 19 arcs. The square nodes are binomial, the triangular node is Poisson, the oval nodes are Gaussian, and the pentagonal node is based on multinomial distributed variables. The gray scale values of the nodes display the contribution levels of the variables (target variable, viral and bacterial pathogens, retrovirus infections, clinical signs, and animal level risk factors). The thickness of the arrow is proportional to their probability, as in classical regressions, where the effect size is almost always reported with a measure of significance. The probability of an arrow in a BN model is the counterpart of the *P*-value in a regression analysis. The percentages are reported in Table 2 under *Support*. This table also shows the regression coefficients and their interpretation. The Confidence Intervals (CIs) are Wald-type CIs. The odds and rate ratios have a simple epidemiological interpretation. If smaller than one, a ratio has a negative effect. Inversely, if larger than one, the effect is positive. This direction of the effect is displayed in the DAG.

**Table 2:** Regression coefficient estimates and 95% Confidence Intervals (CI) with their interpretation and data support (computed with structural MCMC).

**Arc**	**Coefficient**	**95% CI**	**Interpretation**	**Support [%]**
FCV–Vaccinated	0.38	[0.2;0.72]	Odds ratio	70.7
FCV–Gingivostomatitis	8.17	[4.63;14.42]	Odds ratio	100
*C. felis*–URTD	22.20	[3.13;157.59]	Odds ratio	100
*C. felis*–Age	0.33	[0.14;0.77]	Odds ratio	94.9
*M. felis*–FCV	2.69	[1.62;4.48]	Odds ratio	100
*M. felis*–FHV-1	3.00	[1.54;5.58]	Odds ratio	53.2
*M. felis*–Outdoor	0.50	[0.31;0.82]	Odds ratio	59.9
Gingivostomatitis–Pedigree	3.00	[1.74;5.20]	Odds ratio	100
Gingivostomatitis–Age	1.54	[1.19;1.98]	Odds ratio	98.7
URTD–FHV-1	2.69	[1.41;5.14]	Odds ratio	56.3
Outdoor–Pedigree	0.12	[0.07;0.22]	Odds ratio	100
Group size–FCV	1.57	[1.35;1.82]	Rate ratio	100
Group size–*C. felis*	0.61	[0.44;0.83]	Rate ratio	97.2
Group size–*M. felis*	1.26	[1.10;1.45]	Rate ratio	95.3
Group size–*B. bronchiseptica*	2.56	[2.02;3.24]	Rate ratio	100
Group size–Gingivostomatitis	0.77	[0.66;0.90]	Rate ratio	99.1
Group size–URTD	1.27	[1.11;1.46]	Rate ratio	99.2

### 5.8. Interpretation of the Findings

The ABN analysis aims at studying the determinants of the FCV status. Despite using a different framework for the analysis and different datasets, the findings of the present case study are very similar to the initial results presented by Berger et al. (44). In Figure 7, the FCV status is directly associated with the vaccination status, the gingivostomatitis complex, the size of the housing group, and the presence of *M. felis*. The vaccination is negatively associated with the FCV status, with an odds ratio of 0.38 (i.e., the vaccinated cats are less likely to have a positive FCV status) and a supportive probability of 70.7%. The FCV status is positively associated with gingivitis and stomatitis aggregated, with an odds ratio of 8.17 in all MCMC samples. Gingivostomatitis indicates an inflammation of the caudal and buccal oral mucosa and, occasionally, other oral mucosal surfaces. The FCV status is also positively directly associated with the presence of *M. felis*, with an odds ratio of 2.69, with a supportive probability of 100%. Housing cats in large groups is also found to be a risk factor (with a rate ratio of 1.57 present in all MCMC samples).

The original study used a dichotomized variable for group size. In the present case study, we used a Poisson-distributed variable. Based on Figure 5, a zero-inflated or negative binomial may have been a better choice. Unfortunately, these distributions are not available in the abn R package. Interestingly, as was found in the original publication (44), classical signs of URTD (such as nasal discharge, ocular discharge, conjunctivitis, and sneezing) are not found to be directly associated with FCV status. The reduction of the group size and vaccination are the two actionable factors found to be directly associated with the FCV status and are recommended as a measure to control FCV infection. Alternatively, the presence of gingivostomatitis or *M. felis* infection is a strong indicator of an FCV-positive status in a cat. Another nice feature of a BN analysis is the possibility of gaining insights into the relationships with variables other than the targeted one. Beyond the FCV interpretation, one can see that cats with a pedigree are more likely to be vaccinated, less likely to have outdoor access, and less likely to suffer from gingivostomatitis complex compared to non-pedigree cats. The older a cat is, the more likely it is to suffer from gingivostomatitis complex but the less likely it is to be *C. felis*-positive. Figure 7 also shows that a cat's sex with reproductive status, FeLV status, and FIV status are not associated with the rest of the network. However, only five positive cases occur in the dataset for FeLV and FIV status, and so this result should be treated cautiously. Sex and reproductive status seem not to play a role in FCV infection dynamics.

## 6. Discussion and Perspective

This paper introduced BN modeling and highlighted its strengths and weaknesses when applied to complex epidemiological data. We illustrated the key concepts and presented a detailed case study analysis using open data and open code. We hope this will help raise awareness of BN modeling and its potential within the epidemiological community. As a secondary objective, the case study focuses on running a complimentary analysis on an already published dataset about FCV infection among cats in Switzerland. The BN modeling attempts to identify potential determinants of the FCV status and to contrast results with previous results obtained with the standard multivariate approach. The two analyses show very similar results, and the ABN analysis is a convincing alternative to the original statistical approach based on uni- and multi-variate regression models. BN modeling can be seen as a new tool that might be useful for giving additional new insights potentially not captured by classical methods.

BN modeling is typically a *hypothesis-generating* approach used in veterinary epidemiology when very little is known within a research domain. For confirmatory studies, more traditional epidemiological approaches are usually preferred. In machine learning, it is often advisable to follow guidelines for good modeling practices (51, 52). According to the literature, three major points are essential components for good modeling practices:

- Definitions of model objectives and lists of the model assumptions and algorithms used are needed
- Model outputs should be assessed
- The model's outputs must be fully reported.

Good modeling practices are essential to produce robust models, as is transparently reporting possible technical or computational issues and their workarounds. Ideally, we hope to identify and report the single most robust DAG. A significant concern in BN modeling is the tendency to overfit the data and to select overly complicated models that generalize poorly. Albeit being popular and accepted, pruning DAGs using bootstrapping leads to crude choices regarding the possible connections in the model and diminishes the range of possible interpretations. Indeed, an arc is either present or absent. This approach is somewhat rudimentary, considering the massive number of *a priori* networks. Another possible focus would be to seek robust quantification of the connection between variables among the vast number of possible models. In the case study, we emphasize the practical need to account for uncertainty in the final reported DAG through Bayesian model averaging. This methodology is a very active research field that shows encouraging results in closely related domains and seems to be the future of BN modeling. Bayesian model averaging could be very useful in an applied context to avoid reducing the richness of BN modeling to only one single model. Indeed, it allows users to quantify the marginal impact of relationships (arcs in a network) of interest by marginalizing out over networks or nuisance dependencies (i.e., all other possible relationships). Structural MCMC seems to be a very elegant and natural way to quantify the true marginal impact so that one can determine if its magnitude is great enough to consider it as a worthwhile intervention. The main drawback of this technique is its considerable computational demands. The increasing availability of cheap computational resources makes structural Bayesian model averaging feasible for a large variety of studies.

## Data Availability Statement

The datasets analyzed for this study are publicly available and can be found in the abn R package through the command data(“FCV”, package = “abn”).

## Ethics Statement

Ethical review and approval was not required for the animal study because the veterinarians obtained informed consent from the cat owners. All samples were taken as part of a diagnostic workup or for routine testing of FeLV/FIV in healthy cats and all results were provided to the veterinarians and the cat owners; no ethical approval was necessary for this study in compliance with Swiss regulations. Written informed consent was obtained from the owners for the participation of their animals in this study.

## Author Contributions

GK conceived and wrote the manuscript with support from SH, RF, and FL. GK performed the analysis of the case study. GK was co-author of the abn R package and creator and author of the mcmcabn R package. FL was the original creator and author of the ABN method, provided the critical feedback on the structure of the manuscript and on the statistical approach used. RH-L provided the dataset, ensured the possibility of open data, and provided the critical feedback on the manuscript. BW, MM, FB, and RH-L helped with the interpretation of the case study and were the authors of the original publication. BW, FB, and PT provided the critical feedback on the manuscript. RF was the Ph.D. supervisor of GK. RF probed the abn R package and provided useful suggestions that led to numerous improvements on the package. RF provided the input on the statistical framework, model implementation, and findings reporting. SH identified the case study dataset and provided input on the findings interpretation. All authors revised the manuscript.

### Conflict of Interest

The authors declare that the research was conducted in the absence of any commercial or financial relationships that could be construed as a potential conflict of interest.
