# Supplementary material for: Bayesian Network Modeling Applied to Feline Calicivirus Infection Among Cats in Switzerland
Source: Front Vet Sci. 2020 Feb 26;7:73. doi: 10.3389/fvets.2020.00073 (PMC7055399; doi:10.3389/fvets.2020.00073)
Supplement: Supplementary file 1 [file Presentation_1.pdf]

Bayesian Networks modeling applied to Feline Calicivirus infection among cats in Switzerland

Gilles Kratzer [1,*], Fraser I Lewis [2], Barbara Willi [3], Marina L. Meli [4,5], Felicitas S. Boretti [3], Regina Hofmann-Lehmann [4,5], Paul Torgerson [6], Reinhard Furrer [1,7] and Sonja Hartnack [6]

[1] *Department of Mathematics, University of Zurich, CH-8057 Zurich, Switzerland*
[2] *Independent researcher, Utrecht, the Netherlands*
[3] *Clinic for Small Animal Internal Medicine, Vetsuisse Faculty, University of Zurich, CH-8057 Zurich, Switzerland*
[4] *Clinical Laboratory, Department of Clinical Diagnostics and Services, Vetsuisse Faculty, University of Zurich, CH-8057 Zurich, Switzerland*
[5] *Center for Clinical Studies, Vetsuisse Faculty, University of Zurich, CH-8057 Zurich, Switzerland*
[6] *Section of Epidemiology, Vetsuisse Faculty, University of Zurich, CH-8057 Zurich, Switzerland*
[7] *Department of Computational Science, University of Zurich, CH-8057 Zurich, Switzerland*

Correspondence*:
Department of Mathematics, University of Zurich, Winterthurerstrasse 190, CH-8057 Zurich, Switzerland
gilles.kratzer@math.uzh.ch

ANNEX

This section presents the technical details about the class equivalence and the technique used to control for overfitting and perform Bayesian model averaging.

Network equivalence class

This annex aims at presenting one strong limitation of BN models learned with probabilistic methods. A given factorization of the joint probability distribution can be represented by different DAGs (see figure 1) (Pearl, 2000). Reversing some arrows in a given network may leave the probabilistic model unchanged. A probabilistic method can at most learn the so-called Markov equivalence class of a network, which is the network encoding a given model. A Markov class is represented by a partially directed acyclic graph (PDAG), which means that some edges are directed (arrows) and some edge are not directed (the ones leading to equivalent probabilistic models).

Two random variables X and Y are said to be conditionally independent of a set of random variables Z if

$$P(X, Y \mid Z) = P(X \mid Z)P(Y \mid Z). \tag{1}$$

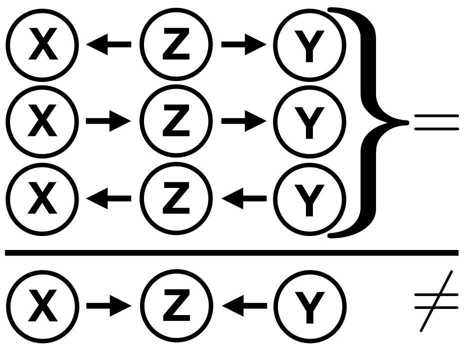

Figure 1. Four basic BN and their equivalence class. The upper networks share the same set of conditional independencies. The v-structure network s derived from a different probabilistic model.

By using the definition of conditional probability, it is possible to compute the conditional independencies implied by the different arrangements presented in figure 1. The conditional independence statements are equal for the three upper networks but different for the v-structure, as shown in (Scutari and Denis, 2014). We use this simple example to highlight the fact that equivalence classes in DAGs exist and that they are a substantial limitation to the interpretation of the arc's direction in a DAG.

Sample size consideration

A recurrent question when modeling epidemiological data using BN models is the number of observations needed for performing a valid inference. This is a complicated issue that does not have a theoretical answer and is hard to be studied with simulation studies. Some theoretical attempts have been made to find lower bound for the required sample size. But they rely on either simplistic settings that cannot be applied in practice or on an untestable set of assumptions. The reason why simulation studies are of little help here is that multiple independent approximations are used in the learning process. For example, the learning algorithm could be heuristic and then leading to approximately best network. The scoring system used, which is data-dependent is necessarily imperfect, and often simulation studies rely on synthetic datasets that could have a biological unrealistic signal-to-noise ratios. Liu et al. (2012) studied the efficiency of the scoring system using real-world datasets of different sample sizes and different network complexity and shows learning performances. This paper contains multiple real-world datasets that could give the reader some ideas of the typical number of data points regarding the network size and complexity used in BN models. No simple metric exists to assess the number of independent data points per model parameter. For example, assuming 200 data points, one dependent variable, and ten independent variables in a standard regression setting, there are ten arcs. One can still estimate 20 arcs without really demanding much more of the data since the joint distribution is decomposed over the nodes, i.e., this is two regressions of ten arcs, rather than one regression of now 20 variables. The FCV dataset has 280 observations, plus 20 imputed data points, and the presented network is made of 15 nodes, 19 arcs with a maximum number of parents per node of seven. This means that the largest regression model estimated in the analysis is made of seven covariates for 280 data points. Sample size is always a concern with BN models. A good practice is then to perform a statistically rigorous model selection process with a bootstrapping step, which is aimed at avoiding spurious structural findings.

Adjustment for overfitting

In machine learning, it is known that automated procedures, such as BN modeling, are usually susceptible to overfitting the data. It is paramount to control for overfitting (Friedman et al., 1999), typically done using parametric (Comin et al., 2019) and non-parametric (Ruchti et al., 2019) bootstrapping. An alternative approach is to use a structural MCMC approach. An algorithm based on a single edge move (deletion, addition or reversal), proposed by Madigan et al. (1995) and Giudici and Castelo (2003), is known as the Monte Carlo Markov Chain Model Choice ($(MC)^3$). The idea of this algorithm is to move from one network to another with respect to their probabilities. The $(MC)^3$ mix poorly and get stuck easily in a Markov class, failing to sample the global landscape of plausible networks efficiently. Updates have been proposed to surpass this weakness (Grzegorczyk and Husmeier, 2008; Su and Borsuk, 2016) by performing more drastic changes in the proposed network in order to pass to another class (i.e., change more than a single arc). Alternatives to the structural MCMC approach are the Gibbs sampler by Goudie and Mukherjee (2016) or the order-MCMC by Friedman and Koller (2003). Recently, an upgraded unbiased order MCMC with an efficient R implementation well adapted to large networks BiDAG available on CRAN has been proposed Kuipers and Moffa (2017). The advantage of the structural MCMC sampler (implemented in R as mcmcabn and available on CRAN) is its transparency with respect to priors used and its compatibility with the type of restrictions imposed in typical ABN analyses. Moreover, this algorithm shows similar results as order MCMC without having a systematic bias.

In order to control for overfitting, a large MCMC run has been performed to sample high scoring networks. Based on this sample, we will prune the best DAG to keep only top supported arcs. As a by-product, we will estimate the support of each arc. Based on a cache of pre-computed scores, four MCMC runs were lunched with 30,000 steps and a thinning factor of 100. A burn-in phase of 5,000 steps was used. The initial DAG is the one with the largest possible score. The global structural prior used is a non-informative prior where parent combinations of all cardinalities are equally likely. The MCMC runs each integrated the two implemented drastic changes in 1.5% of the time. This probability should be large enough to ensure a good sampling across the equivalence classes but not too high to avoid introducing a bias and extra computation time. The model constraints regarding the nodes *Sex, Age* and *Pedigree* are contained in the pre-computed cache of scores, and the moves are restricted to the ones compatible with the given structural constraints. Multiple chain convergence has been checked with potential scale reduction factors (0.999 with an upper $CI_{95\%}$ of 1) (Brooks and Gelman, 1998). The final MCMC sample is obtained as the union of the four independent thinned chains. The global acceptance rate is 4.5%. The parameter `mcmc.scheme` encodes in that order: the number of MCMC steps, the thinning factor and the number of burn-in steps. Then 250 steps are returned by each chain. The code below allows to perform one search.

```
R> mcmc.out <- mcmcabn(score.cache = cache,
+    score = "bic",
+    data.dists = dists,
+    max.parents = 7,
+    mcmc.scheme = c(25000,99,5000),
+    start.dag = dag,
+    prob.rev = 0.015,
```

```
+    prob.mbr = 0.015,
+    prior.choice = 1)
```

When the dimension of the dataset is too big for an exact search that covers most real-world datasets, it is possible to choose `start.dag = "hc"`, which performs a heuristic search before starting the MCMC procedure. An alternative is to separately optimize a DAG to get a single best model and then feed it into the MCMC sampler. In this case study, computing the scores takes 20 minutes using `buildscorecache()` with a desk computer. The best model takes about 10 minutes to be computed using `mostprobable()`. One MCMC run takes approximately 30 hours to be computed. The multiple chains can be computed in parallel.

Figure 2 shows the trace plot of the network's scores for the four MCMC runs, starting from the highest scoring DAG. The dashed red line is the maximum score (as expected −6403.01). The densities on the right represent the frequencies of the DAG moves for each MCMC run. The grey area is the burn-in phase (discarded from all further computations). As expected, the $(\mathrm{MC})^3$ algorithm efficiently samples the landscape of high-scoring DAGs. One can see very good mixing in the MCMC runs. The inset plot displays a trace plot of two MCMC runs starting from random graphs. They lead to very similar results at the expense of a much larger computation time (one week per chain). The potential scale reduction factor is 1.02 with an upper $\mathrm{CI}_{95\%}$ of 1.1, two chains of 150,000 steps, 5,000 steps of burn-in and a thinning of 100.

A simple diagnostic to assess the amount of overfitting in the modeling process can be done by plotting the distribution of the number of arcs in the MCMC sample. Figure 3 shows the number of arcs in the MCMC sample. The highest score's BN has 20 arcs, which is above the mean of the distribution and thus a strong indicator of overfitting.

Based on the MCMC sample, we can compute the individual arc support. Table 1 shows the individual arc supportive probability within the MCMC sample. To produce a majority consensus, DAG 1 prunes the arcs supported in less than 50% of the MCMC sample. Among the 225 possible associations, only four scores between 20% and 80% support probability. This indicates that the MCMC procedure, thresholded at 50%, produces a well defined consensus DAG. The majority consensus DAG is reported in the main paper, where the arc's probability is represented by the thickness of the arcs.

Figure 4 shows the frequency of the 22 most represented DAGs within the MCMC sample. They collectively represent 20% of the total generated DAGs. The main incitement to perform Bayesian model averaging to account for uncertainty in BN models, as one can see, is the huge diversity in terms of model characteristics (the number of arcs, the scores and the distance (i.e., the number of changes) between DAGs) in this figure. Indeed, reporting a uniquely adjusted DAG looks extremely elementary, given the number of possible models. Model averaging is a very promising research topic that already shows impressive results (Moffa et al., 2017; Kuipers et al., 2018, 2019). The case study aims at keeping this richness while reporting arcs with supportive probabilities as well as simple structural queries.

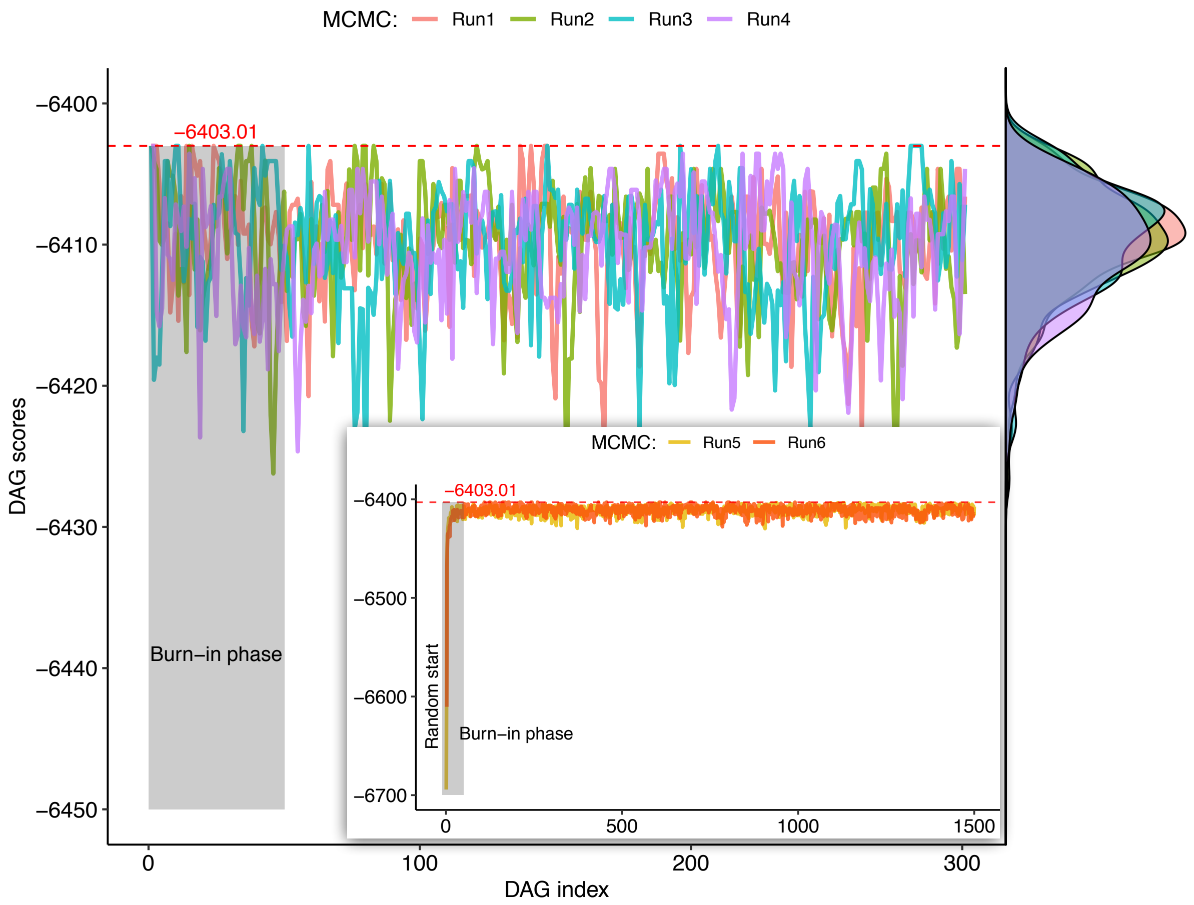

Figure 2. Trace plot of the network's scores from four MCMC chains, starting from the highest scoring DAG. The dashed red line is the maximum reached score (as expected -6403.01) found with the `mostprobable()` function. The density on the right represents the occurrence of the DAG scores within each chain. The dashed area is the burn-in phase. The inset panel displays a trace plot of two MCMC chains starting with random DAGs.

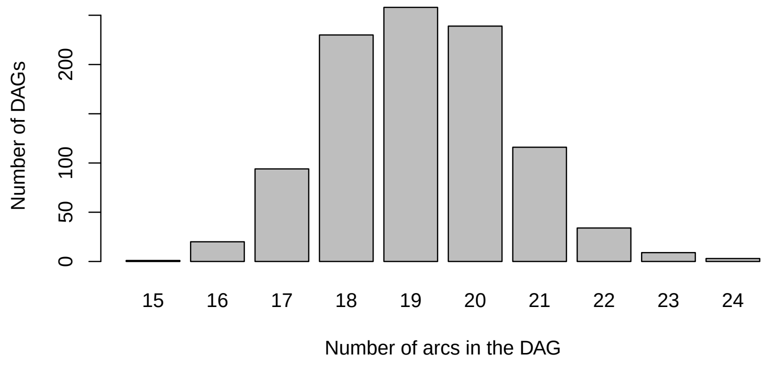

Figure 3. Histogram of the distribution of the number of arcs in the MCMC sample. The highest score's BN has 20 arcs, which is more than the mode of the distribution.

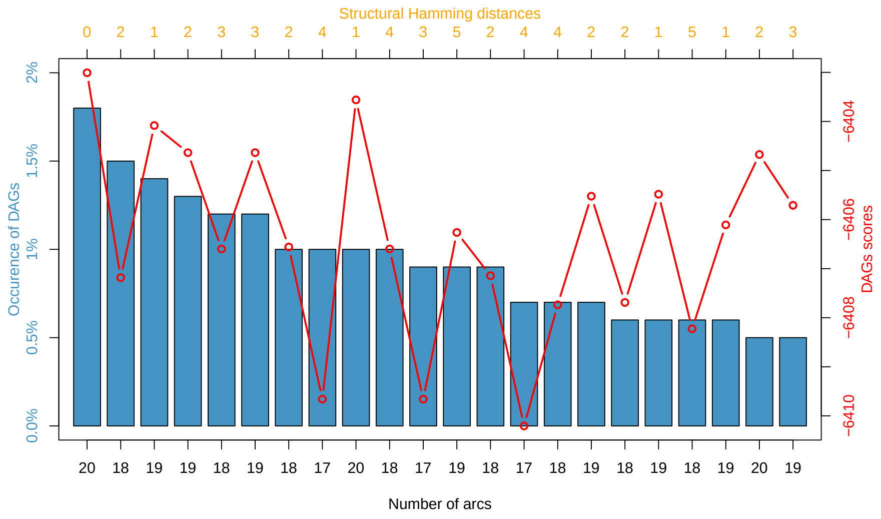

Figure 4. Distribution of the 20% most frequent DAGs with their frequencies in the MCMC sample. The x-axis displays the total number of arcs of the networks. The y-axis displays the percentages within the MCMC sample. The second y-axis (in red) shows the network's scores. The second x-axis (in orange) dispays the Structural Hamming Distances (SHD), which counts how many arcs differ between the most represented DAG and the others. The SHD is a proxy to compare how different two DAGs are.

Structural queries

The structural queries presented in the case study can be computed in the mcmcabn R package using following code:

What is the probability that the classical signs of URTD (nasal discharge, ocular discharge, conjunctivitis and sneezing) are NOT associated with the FCV status?

```
R> 1 - query(mcmcabn = mcmc.out,
+    formula = ~URTD|FCV) -
+    query(mcmcabn = mcmc.out,
+    formula = ~FCV|URTD)
 [1]  0.997
```

What is the probability of the gingivostomatitis complex being directly associated to the FCV status if the vaccination status is NOT?

```
R> query(mcmcabn = mcmc.out,
+    formula = ~FCV|Gingivostomatitis-FCV|Vaccinated) +
+    query(mcmcabn = mcmc.out,
+    formula = ~Gingivostomatitis|FCV-FCV|Vaccinated) +
+    query(mcmcabn = mcmc.out,
+    formula = ~Gingivostomatitis|FCV-Vaccinated|FCV) +
+    query(mcmcabn = mcmc.out.,
+    formula = ~FCV|Gingivostomatitis-Vaccinated|FCV)
 [1]  0.5
```

Table 1. Table of the percentage of the individual arcs supported by the MCMC sample. The children nodes are listed in the rows and the parent nodes are listed in the columns.

	FCV	FHV-1	*C. felis*	*M. felis*	*B. bronchi septica*	FeLV	FIV	Gingivo stomatitis	URTD	Vaccinated	Pedigree	Outdoor	Sex	Group Size	Age
FCV	NA	0.0	0.0	0.0	0.7	1.0	0.7	100.0	0.2	70.7	0.2	0.6	7.8	0.0	1.5
FHV-1	0.0	NA	0.0	45.6	2.5	0.1	0.9	0.7	28.4	0.3	0.1	0.3	0.0	0.0	0.3
C. felis	0.3	0.8	NA	0.1	0.5	0.2	0.6	0.2	100.0	0.1	0.1	0.0	0.0	0.0	94.9
M. felis	100.0	53.2	0.4	NA	0.2	0.1	0.2	0.2	6.0	0.0	0.7	59.9	0.0	0.0	0.1
B. bronchiseptica	0.3	2.3	0.6	0.4	NA	0.6	0.1	0.0	4.0	0.1	0.0	0.4	0.0	0.0	0.4
FeLV	0.5	0.0	0.2	0.1	0.9	NA	0.5	0.4	0.1	0.2	1.3	6.1	0.0	0.1	0.0
FIV	0.4	1.1	0.1	0.0	0.2	0.0	NA	0.1	0.5	0.9	0.0	0.8	0.0	0.3	0.3
Gingivostomatitis	0.0	0.3	0.0	0.0	0.1	1.3	0.0	NA	1.2	0.0	100.0	0.1	0.0	0.0	98.7
URTD	0.1	56.3	0.0	11.0	2.6	0.3	0.6	22.8	NA	2.7	0.3	0.0	0.0	0.0	6.5
Vaccinated	3.1	0.2	0.3	0.3	0.1	0.0	0.9	0.3	3.4	NA	69.5	16.0	0.5	0.0	9.4
Pedigree	0.0	0.0	0.0	0.0	0.0	0.0	0.0	0.0	0.0	0.0	NA	0.0	0.0	0.0	0.0
Outdoor	0.4	0.0	0.1	2.2	0.3	2.2	0.8	0.3	0.0	0.7	100.0	NA	0.0	0.0	1.4
Sex	0.0	0.0	0.0	0.0	0.0	0.0	0.0	0.0	0.0	0.0	0.0	0.0	NA	0.0	0.0
Group Size	100.0	0.0	97.2	95.3	100.0	0.2	0.3	99.1	99.2	0.2	0.0	100.0	0.0	NA	0.0
Age	0.0	0.0	0.0	0.0	0.0	0.0	0.0	0.0	0.0	0.0	0.0	0.0	0.0	0.0	NA

REFERENCES

Brooks, S. P. and Gelman, A. (1998). General methods for monitoring convergence of iterative simulations. *Journal of Computational and Graphical Statistics* 7, 434–455

Comin, A., Jeremiasson, A., Kratzer, G., and Keeling, L. (2019). Revealing the structure of the associations between housing system, facilities, management and welfare of commercial laying hens using additive Bayesian networks. *Preventive Veterinary Medicine* 164, 23–32

Friedman, N., Goldszmidt, M., and Wyner, A. (1999). Data analysis with Bayesian networks: A bootstrap approach. In *Proceedings of the Fifteenth Conference on Uncertainty in Artificial Intelligence* (Morgan Kaufmann Publishers Inc.), 196–205

Friedman, N. and Koller, D. (2003). Being Bayesian about network structure. A Bayesian approach to structure discovery in Bayesian networks. *Machine Learning* 50, 95–125

Giudici, P. and Castelo, R. (2003). Improving Markov chain Monte Carlo model search for data mining. *Machine Learning* 50, 127–158

Goudie, R. J. and Mukherjee, S. (2016). A Gibbs sampler for learning DAGs. *The Journal of Machine Learning Research* 17, 1032–1070

Grzegorczyk, M. and Husmeier, D. (2008). Improving the structure mcmc sampler for Bayesian networks by introducing a new edge reversal move. *Machine Learning* 71, 265

Kuipers, J. and Moffa, G. (2017). Partition MCMC for inference on acyclic digraphs. *Journal of the American Statistical Association* 112, 282–299

Kuipers, J., Moffa, G., Kuipers, E., Freeman, D., and Bebbington, P. (2019). Links between psychotic and neurotic symptoms in the general population: An analysis of longitudinal British national survey data using directed acyclic graphs. *Psychological Medicine* 49, 388–395

Kuipers, J., Thurnherr, T., Moffa, G., Suter, P., Behr, J., Goosen, R., et al. (2018). Mutational interactions define novel cancer subgroups. *Nature Communications* 9, 4353

Liu, Z., Malone, B., and Yuan, C. (2012). Empirical evaluation of scoring functions for Bayesian network model selection. In *BMC Bioinformatics* (BioMed Central), vol. 13, S14

Madigan, D., York, J., and Allard, D. (1995). Bayesian graphical models for discrete data. *International Statistical Review/Revue Internationale de Statistique* 63, 215–232

Moffa, G., Catone, G., Kuipers, J., Kuipers, E., Freeman, D., Marwaha, S., et al. (2017). Using directed acyclic graphs in epidemiological research in psychosis: An analysis of the role of bullying in psychosis. *Schizophrenia Bulletin* 43, 1273–1279

Pearl, J. (2000). *Causality: Models, Reasoning and Inference*, vol. 29 (Springer-Verlag)

Ruchti, S., Kratzer, G., Furrer, R., Hartnack, S., Würbel, H., and Gebhardt-Henrich, S. G. (2019). Progression and risk factors of pododermatitis in part-time group housed rabbit does in Switzerland. *Preventive Veterinary Medicine* 166, 56–64

Scutari, M. and Denis, J.-B. (2014). *Bayesian Networks: with Examples in R* (Chapman and Hall/CRC)

Su, C. and Borsuk, M. E. (2016). Improving structure MCMC for Bayesian networks through Markov blanket resampling. *The Journal of Machine Learning Research* 17, 4042–4061
