# Supplementary material for: Bayesian Network Modeling Applied to Feline Calicivirus Infection Among Cats in Switzerland
Source: Front Vet Sci. 2020 Feb 26;7:73. doi: 10.3389/fvets.2020.00073 (PMC7055399; doi:10.3389/fvets.2020.00073)
Supplement: Supplementary file 2 [file Data_Sheet_1.docx]

##---------------------------------------------------------------------

## R code to reproduce the findings from the article entitled:

## "Bayesian Networks modeling applied to Feline Calicivirus infection among cats in Switzerland"

## author: gilles.kratzer@gmail.com

## WARNING: this script takes more than 40 hours to be executed!!!

## ---------------

## library loading

library("readxl")

library(lubridate)

library(abn)

library(mice)

library(mcmcabn)

library(kableExtra)

library(coda)

library(ggplot2)

library(ggpubr)

library(reshape2)

library(cowplot)

library(abind)

library(grid)

##cleaning

rm(list = ls()) # clear objects

graphics.off() # close graphics windows

##data save

dat.save <- c("../Output/Rdat/")

##output

out.save <- c("../Output/")

##########################################################################

############################## LOADING DATA ##############################

##########################################################################

dta <- data("FCV", package = "abn")

##########################################################################

############################## ABN analysis ##############################

##########################################################################

dists <- list(FCV = "binomial",

FHV_1 = "binomial",

C_felis = "binomial",

M_felis = "binomial",

GroupSize = "poisson",

Sex = "multinomial",

Pedigree = "binomial",

Age = "gaussian",

B_bronchiseptica = "binomial",

Vaccinated = "binomial",

Outdoor = "binomial",

FeLV = "binomial",

FIV = "binomial",

Gingivostomatitis = "binomial",

URTD = "binomial")

dists <- dists[names(dta)]

dists_shame <-list(FCV = "gaussian",

FHV_1 = "gaussian",

C_felis = "gaussian",

M_felis = "gaussian",

GroupSize = "gaussian",

Sex = "gaussian",

Pedigree = "gaussian",

Age = "gaussian",

B_bronchiseptica = "gaussian",

Vaccinated = "gaussian",

Outdoor = "gaussian",

FeLV = "gaussian",

FIV = "gaussian",

Gingivostomatitis = "gaussian",

URTD = "gaussian")

dists_shame <- dists_shame[names(dta)]

## score search

bic.values <- aic.values <- mdl.values <- vector(length = 11)

# for loop for discovering needed network complexity

for (i in 1:11) {

max.par <- i

# construction of the score cache

mycache <- buildscorecache(data.df = mice_output,

data.dists = dists,

dag.banned = ~Sex|.+Age|.+Pedigree|.,

max.parents = max.par,method = "mle")

# optimal dag with AIC

dag <- mostprobable(score.cache = mycache,score = "bic")

fabn <- fitabn(object = dag,method = "mle")

bic.values[i] <- fabn$bic

# optimal dag with AIC

dag <- mostprobable(score.cache = mycache,score = "aic")

fabn <- fitabn(object = dag,method = "mle")

aic.values[i] <- fabn$aic

# optimal dag with MDL

dag <- mostprobable(score.cache = mycache,score = "mdl")

fabn <- fitabn(object = dag,method = "mle")

mdl.values[i] <- fabn$mdl

}

pdf(file = paste0(out.save,"bic.pdf"))

plot(1:max.par, -bic.values, xlab = "Parent max limit", ylab = "Network score (BIC)",

type = "b", col="red", ylim=range(-bic.values))

abline(v=which(-bic.values==max(-bic.values))[1], col="grey", lty=2, lwd=4)

dev.off()

# BIC 7 parents needed

pdf(file = paste0(out.save,"aic.pdf"))

plot(1:max.par, -aic.values, xlab = "Parent max limit", ylab = "Network score (AIC)",

type = "b", col="red", ylim=range(-aic.values))

abline(v=which(-aic.values==max(-aic.values))[1], col="grey", lty=2, lwd=4)

dev.off()

# AIC 10 parents needed

## ----------------------------

## Max.parent versus score plot

pdf(file = paste0(out.save,"scores.pdf"),width = 7,height = 4)

plot(1:max.par, 1/(-bic.values/max(-bic.values)), xlab = "Max. parent limit", ylab = "Max. network score [%]",

type = "b", col="red", ylim=range(0.975,1),yaxt="n",xaxt="n")

axis(2, at=c(0.975,0.985,0.995,1), labels=c(97.5,98.5,99.5,100))

axis(1, at=1:11, labels=1:11)

abline(v=which(-bic.values==max(-bic.values))[1]+0.1, col="red", lty=2, lwd=4)

lines(1:max.par, 1/(-aic.values/max(-aic.values)),

type = "b", col="blue", ylim=range(-aic.values))

abline(v=which(-aic.values==max(-aic.values))[1], col="blue", lty=2, lwd=4)

lines(1:max.par, 1/(-mdl.values/max(-mdl.values)),

type = "b", col="green", ylim=range(-mdl.values))

abline(v=which(-mdl.values==max(-mdl.values))[1]-0.1, col="green", lty=2, lwd=4)

abline(h=1, col="gray", lty=2, lwd=4)

legend(x ="bottomright", legend = c("AIC","BIC", "MDL"),col = c("blue", "red", "green"),lwd=4,bty = "n")

dev.off()

plot(dag)

dev.off()

## --------------------------

## adjustment for overfitting

mycache <- buildscorecache(data.df = mice_output,

data.dists = dists,

dag.banned = ~Sex|.+Age|.+Pedigree|.,

max.parents = 7,method = "mle")

dag <- mostprobable(score.cache = mycache,score = "bic")

plotabn(dag.m = dag$dag,data.dists = dists)

save(mice_output, dists,mycache,dag,file = paste0(dat.save,"bootstrapping_input.Rdat"))

##-------------------

## Bootstrapping step

## ------------------

start.time <- Sys.time()

mcmc.out.1 <- mcmcabn(score.cache = mycache,

score = "bic",

data.dists = dists,

max.parents = 7,

mcmc.scheme = c(25000,0,0),

seed = 5637,

verbose = TRUE,

start.dag = dag$dag,

prob.rev = 0.03,

prob.mbr = 0.03,

prior.choice = 1)

end.time <- Sys.time()

time.taken <- end.time - start.time

start.time <- Sys.time()

mcmc.out.2 <- mcmcabn(score.cache = mycache,

score = "bic",

data.dists = dists,

max.parents = 7,

mcmc.scheme = c(25000,0,0),

seed = 4896,

verbose = TRUE,

start.dag = dag$dag,

prob.rev = 0.03,

prob.mbr = 0.03,

prior.choice = 1)

end.time <- Sys.time()

time.taken <- end.time - start.time

start.time <- Sys.time()

mcmc.out.3 <- mcmcabn(score.cache = mycache,

score = "bic",

data.dists = dists,

max.parents = 7,

mcmc.scheme = c(25000,0,0),

seed = 41894896,

verbose = TRUE,

start.dag = dag$dag,

prob.rev = 0.03,

prob.mbr = 0.03,

prior.choice = 1)

end.time <- Sys.time()

time.taken <- end.time - start.time

start.time <- Sys.time()

mcmc.out.4 <- mcmcabn(score.cache = mycache,

score = "bic",

data.dists = dists,

max.parents = 7,

mcmc.scheme = c(25000,0,0),

seed = 23146,

verbose = TRUE,

start.dag = dag$dag,

prob.rev = 0.03,

prob.mbr = 0.03,

prior.choice = 1)

end.time <- Sys.time()

time.taken <- end.time - start.time

##--------------------------------------

##analysis

##--------------------------------------

## gelman

list.mc <- mcmc.list(mcmc(mcmc.out.1$scores[c(TRUE,rep(FALSE,99))]),mcmc(mcmc.out.2$scores[c(TRUE,rep(FALSE,99))]) ,mcmc(mcmc.out.3$scores[c(TRUE,rep(FALSE,99))]),mcmc(mcmc.out.4$scores[c(TRUE,rep(FALSE,99))]))

gelman.diag(x = list.mc,autoburnin = TRUE)

gelman.plot(list.mc)

## traceplot

fabn <-fitabn(dag.m = dag,data.df = mice_output,data.dists = dists, method = "mle") #

max.score <- -6403.01 #-fabn$bic

dta <- data.frame(mcmc.out.1[2:4],mcmc.out.2[2:4],mcmc.out.3[2:4],mcmc.out.4[2:4])

dta <- dta[,c(1,4,7,10)]

dta <- dta[c(TRUE,rep(FALSE,99)), ]

names(dta) <- c("Run1","Run2","Run3","Run4")

dta$X <- (1:length(dta$Run1))

dta <- melt(dta, "X")

# Create a text

original_plot <- ggplot(data = dta, aes_string(x = "X", y="value", color = "variable")) +

geom_line(alpha = 0.8,lwd=1.1) +

geom_hline(yintercept = max.score,linetype = "dashed", color = "red", alpha = 1) +

geom_text(aes(25, max.score, label = round(max.score,digits = 2), vjust = -0.5), color = "red", check_overlap = TRUE) +

labs(x = "DAG index", y = "DAG scores", colour = "MCMC:") +

theme_pubr() + ylim(-6450,-6400) +

annotate("rect", xmin=0, xmax=50, ymin=-6450, ymax=max.score,

alpha = .3) +

geom_text(aes(25, -6440, label = "Burn-in phase", vjust = -0.5), color = "black", check_overlap = TRUE)

# Plot

y_density <- axis_canvas(original_plot, axis = "y", coord_flip = TRUE) +

geom_density(data = dta, aes_string(x = "value",fill = "factor(variable)"), alpha = 0.5) +

coord_flip()

# create the combined plot

ggsave(paste0(out.save,"mcmc.pdf"),

plot = ggdraw(insert_yaxis_grob(plot = original_plot, grob = y_density, position = "right")),

width = 9,height = 7)

dev.off()

##best dag trimmed for controlling overfitting

mcmc.out.1$dags <- mcmc.out.1$dags[,,-(1:5000)]

mcmc.out.2$dags <- mcmc.out.2$dags[,,-(1:5000)]

mcmc.out.3$dags <- mcmc.out.3$dags[,,-(1:5000)]

mcmc.out.4$dags <- mcmc.out.4$dags[,,-(1:5000)]

mcmc.out <- abind(mcmc.out.1$dags[,,c(TRUE,rep(FALSE,99))],mcmc.out.2$dags[,,c(TRUE,rep(FALSE,99))],mcmc.out.3$dags[,,c(TRUE,rep(FALSE,99))],mcmc.out.4$dags[,,c(TRUE,rep(FALSE,99))])

mcmc.out.1$scores <- mcmc.out.1$scores[-(1:5000)]

mcmc.out.2$scores <- mcmc.out.2$scores[-(1:5000)]

mcmc.out.3$scores <- mcmc.out.3$scores[-(1:5000)]

mcmc.out.4$scores <- mcmc.out.4$scores[-(1:5000)]

mcmc.out.score <- list(mcmc.out.1$scores[c(TRUE,rep(FALSE,99))],mcmc.out.2$scores[c(TRUE,rep(FALSE,99))],mcmc.out.3$scores[c(TRUE,rep(FALSE,99))],mcmc.out.4$scores[c(TRUE,rep(FALSE,99))])

dag.mcmc <- apply(mcmc.out, 1:2, mean)

colnames(dag.mcmc) <- rownames(dag.mcmc) <- names(dists)

dag.boot.50 <- dag.mcmc

dag.boot.50[dag.mcmc>0.5]<-1

dag.boot.50[dag.mcmc<=0.5]<-0

plotabn(dag.m = dag.boot.50,data.dists = dists)

fabn <-fitabn(dag.m = dag.boot.50,data.df = mice_output,data.dists = dists, method = "mle")

fabn$bic

infoDag(dag.boot.50)

h <- apply(mcmc.out, 3, sum)

pdf(file = paste0(out.save,"arcs.pdf"),width = 7,height = 4)

barplot(table(h),col = "grey",xlab = "Number of arcs in the DAG", ylab = "Number of DAGs")

dev.off()

u.list.dag <- unique.array(x = mcmc.out,MARGIN = 3)

num_100 <- apply(X = u.list.dag, MARGIN = 3, FUN = function(x){

sum(apply(X = mcmc.out,MARGIN = 3,FUN = function(y){

if(identical(x,y)){1}else{0}

}))

})

max(which((cumsum(sort(num_100,decreasing = FALSE)))/1000<0.80,arr.ind = TRUE))

sort(num_100,decreasing = TRUE)[1:22]

order(num_100,decreasing = TRUE)[1:22]

plot(sort(num_100,decreasing = TRUE)[1:22]/sum(num_100),type = "l")

##plot

scores.dags <- vector(length = 22)

num.arcs <- vector(length = 22)

shd <- vector(length = 22)

for(i in 1:22){

dag <- u.list.dag[,,order(num_100,decreasing = TRUE)[i]]

colnames(dag) <- rownames(dag) <- names(dists)

fabn <- fitabn(dag.m = dag,data.df = mice_output,data.dists = dists,method = "mle")

scores.dags[i] <- -fabn$bic

num.arcs[i] <- sum(dag)

shd[i] <- compareDag(ref = u.list.dag[,,order(num_100,decreasing = TRUE)[1]],u.list.dag[,,order(num_100,decreasing = TRUE)[i]])$`Hamming-distance`

}

pdf(file = paste0(out.save,"mcmc_diversity.pdf"),width = 10,height = 6)

par(mar=c(5,4,4,4))

plot(1:22, sort(num_100,decreasing = TRUE)[1:22], type = 'n',ylab = "",xlab = "Number of arcs",xaxt="n",yaxt="n", ylim = c(0,20))

axis(2,at = c(0, 5,10,15, 20),labels = c("0.0%","0.5%","1%", "1.5%", "2%"),col.axis = "#4393C3")

mtext("Occurence of DAGs", side=2, line=2, col="#4393C3")

rect(1:22 - .4, 0, 1:22 + .4, sort(num_100,decreasing = TRUE)[1:22], col = '#4393C3')

par(new = TRUE)

plot(x = 1:22,y = scores.dags,col="red", type = 'b', lwd=2, axes = FALSE, xlab = "",ylab="")

axis(4, col.axis = 'red')

mtext("DAGs scores", side=4, line=2, col="red")

axis(1, col.axis = 'black',at = 1:22,labels = num.arcs)

axis(3, col.axis = 'orange',at = 1:22,labels = shd)

mtext("Structural Hamming distances", side=3, line=2, col="orange")

dev.off()

options(digits=2)

mcmc.out.query<- list(dags=(mcmc.out),data.dist=dists,scores=mcmc.out.score)

1.0-query(mcmcabn = mcmc.out.query,formula = ~URTD|FCV) -

query(mcmcabn = mcmc.out.query,formula= ~FCV|URTD)

query(mcmcabn = mcmc.out.query,formula = ~FCV|Gingivostomatitis-FCV|Vaccinated) +

query(mcmcabn = mcmc.out.query, formula = ~Gingivostomatitis|FCV-FCV|Vaccinated) +

query(mcmcabn = mcmc.out.query, formula = ~Gingivostomatitis|FCV-Vaccinated|FCV) +

query(mcmcabn = mcmc.out.query,formula = ~FCV|Gingivostomatitis-Vaccinated|FCV)

query(mcmc.out.query)*100

## ----------------------------

## stepwise AIC model selection

model.FCV <- glm(formula = FCV~FHV_1+C_felis+M_felis+GroupSize+Sex+Pedigree+Age+B_bronchiseptica+Vaccinated+Outdoor+FeLV+FIV+Gingivostomatitis+URTD,family = binomial(),data = mice_output)

#AIC

mod.prunned <- stepAIC(model.FCV,direction = "both")

mod.prunned$anova

dag.aic <- plotabn(dag.m = ~FCV|Gingivostomatitis:M_felis:GroupSize:Sex:Outdoor:FIV:Vaccinated,data.dists = dists_shame,plot = FALSE)

#BIC

null <- glm(formula = FCV ~ 1, family = binomial(),data = mice_output)

full <- model.FCV

step(null, scope = list(lower=null,upper=full),

direction="both", criterion = "BIC",k = log(300))

dag.bic <- plotabn(dag.m = ~FCV|Gingivostomatitis:M_felis:Vaccinated,data.dists = dists_shame, plot = FALSE)

#############################################################################################################

######################################### Reporting of results ##############################################

#############################################################################################################

#data description

mm <- data.frame(Variable = colnames(mice_output),

Meaning = c("presence of Feline Calici Virus (0/1)",

"presence of Feline Herpes Virus 1 (0/1)",

"presence of chlamydia felis (0/1)",

"presence of mycoplasma felis (0/1)",

"presence of bordetella bronchispetica (0/1)",

"feline leukosis virus (0/1)",

"feline immunodeficiency virus (0/1)",

"Gingivostomatitis complex (0/1)",

"URTD complex (upper respiratory complex) (0/1)",

"vaccination status (0/1)",

"pedigree (0/1)",

"outdoor access (0/1)",

"sex (m,mk,w,wk)",

"number of cats in the group (cont.)",

"age in year (cont.)"))

kable(mm, row.names = FALSE, digits = 2, align = "ll", "latex") %>%

kable_styling(latex_options = "striped", full_width = F, position = "left")

## -------------------------

## Exploratory Data Analysis

pdf(file = paste0(out.save,"eda.pdf"))

par(mfrow=c(5,3), mar=c(2,4,1.5,1))

xx <- barplot(table(mice_output$FCV)/300, ylim=c(0,1.2), main="FCV", ylab="proportion", col.main = "gray50")

text(x = xx, y = table(mice_output$FCV)/300, label = table(mice_output$FCV), pos = 3, cex = 0.8, col = "red")

xx <- barplot(table(mice_output$FHV_1)/300, ylim=c(0,1.2), main="FHV-1", ylab="proportion", col.main = "gray50")

text(x = xx, y = table(mice_output$FHV_1)/300, label = table(mice_output$FHV_1), pos = 3, cex = 0.8, col = "red")

xx <- barplot(table(mice_output$FeLV)/300, ylim=c(0,1.2), main="FeLV", ylab="proportion", col.main = "gray50")

text(x = xx, y = table(mice_output$FeLV)/300, label = table(mice_output$FeLV), pos = 3, cex = 0.8, col = "red")

xx <- barplot(table(mice_output$FIV)/300, ylim=c(0,1.2), main="FIV", ylab="proportion", col.main = "gray50")

text(x = xx, y = table(mice_output$FIV)/300, label = table(mice_output$FIV), pos = 3, cex = 0.8, col = "red")

xx <- barplot(table(mice_output$M_felis)/300, ylim=c(0,1.2), main=expression( italic("Mycoplasma felis")), ylab="proportion", col.main = "gray50")

text(x = xx, y = table(mice_output$M_felis)/300, label = table(mice_output$M_felis), pos = 3, cex = 0.8, col = "red")

xx <- barplot(table(mice_output$C_felis)/300, ylim=c(0,1.2), main=expression( italic("Chlamydia felis")), ylab="proportion", col.main = "gray50")

text(x = xx, y = table(mice_output$C_felis)/300, label = table(mice_output$C_felis), pos = 3, cex = 0.8, col = "red")

xx <- barplot(table(mice_output$B_bronchiseptica)/300, ylim=c(0,1.2), main=expression( italic("Bordetella bronchiseptica")), ylab="proportion", col.main = "gray50")

text(x = xx, y = table(mice_output$B_bronchiseptica)/300, label = table(mice_output$B_bronchiseptica), pos = 3, cex = 0.8, col = "red")

xx <- barplot(table(mice_output$URTD)/300, ylim=c(0,1.2), main="URTD", ylab="proportion", col.main = "gray50")

text(x = xx, y = table(mice_output$URTD)/300, label = table(mice_output$URTD), pos = 3, cex = 0.8, col = "red")

xx <- barplot(table(mice_output$Gingivostomatitis)/300, ylim=c(0,1.2), main="Gingivostomatitis", ylab="proportion", col.main = "gray50")

text(x = xx, y = table(mice_output$Gingivostomatitis)/300, label = table(mice_output$Gingivostomatitis), pos = 3, cex = 0.8, col = "red")

xx <- barplot(table(mice_output$Pedigree)/300, ylim=c(0,1.2), main="Pedigree", ylab="proportion", col.main = "gray50")

text(x = xx, y = table(mice_output$Pedigree)/300, label = table(mice_output$Pedigree), pos = 3, cex = 0.8, col = "red")

xx <- barplot(table(mice_output$Vaccinated)/300, ylim=c(0,1.2), main="Vaccination status", ylab="proportion", col.main = "gray50")

text(x = xx, y = table(mice_output$Vaccinated)/300, label = table(mice_output$Vaccinated), pos = 3, cex = 0.8, col = "red")

xx <- barplot(table(mice_output$Outdoor)/300, ylim=c(0,1.2), main="Outdoor", ylab="proportion", col.main = "gray50")

text(x = xx, y = table(mice_output$Outdoor)/300, label = table(mice_output$Outdoor), pos = 3, cex = 0.8, col = "red")

xx <- barplot(table(mice_output$Sex)/300, ylim=c(0,1.2), main="Sex", ylab="proportion", col.main = "gray50")

text(x = xx, y = table(mice_output$Sex)/300, label = table(mice_output$Sex), pos = 3, cex = 0.8, col = "red")

hist(mice_output$GroupSize, xlab="", main="Group size",prob=TRUE,col="grey",border="white", col.main = "gray50",xlim = c(1,18), ylim = c(0,0.6),breaks = 18)

lines(density(mice_output$GroupSize),lwd=1.5)

hist(mice_output$Age, xlab="", main="Age",prob=TRUE,col="grey",border="white", col.main = "gray50", ylim = c(0,0.6))

lines(density(mice_output$Age),lwd=1.5)

dev.off()

## dag final

tographviz(dag.m = dag.boot.50,data.df = mice_output,data.dists = dists,outfile = "consensus_dag")

fabn <-fitabn(dag.m = dag.boot.50,data.df = mice_output,data.dists = dists, method = "mle")

links <- query(mcmcabn = mcmc.out.100000)

links[links<=0.5]<-0

## DAGs AIC / BIC

#stepwise

tographviz(dag.m = dag.aic,data.df = mice_output,data.dists = dists_shame,outfile = "aic_dag")

tographviz(dag.m = dag.bic,data.df = mice_output,data.dists = dists_shame,outfile = "bic_dag")

##aic 10 parents

##bic 7 parents

mycache <- buildscorecache(data.df = mice_output,

data.dists = dists,

dag.banned = ~Sex|.+Age|.+Pedigree|.,

max.parents = 10,method = "mle")

dag.aic.10parents <- mostprobable(score.cache = mycache,score = "aic")

tographviz(dag.m = dag.aic.10parents$dag,data.df = mice_output,data.dists = dists_shame,outfile = "aic_dag_8parents")

dag.bic.7parents <- mostprobable(score.cache = mycache,score = "bic")

tographviz(dag.m = dag.bic.7parents$dag,data.df = mice_output,data.dists = dists_shame,outfile = "bic_dag_6parents")

##------

##scores

fabn.aic <- fitabn(dag.m = dag.aic.10parents$dag,data.df = mice_output,data.dists = dists,method = "mle")

fabn.bic <- fitabn(dag.m = dag.bic.7parents$dag,data.df = mice_output,data.dists = dists,method = "mle")

plotabn(dag.m = dag.bic.7parents$dag,data.dists = dists)

##-------------

##save results:

save(file = paste0(dat.save,"FCV_compute.Rdata"),mycache,dists,aic.values,bic.values,mdl.values,dag.aic.10parents,dag.bic.7parents,mice_output,fabn.aic, fabn.bic)

## EOF
